# High-resolution time-series transcriptomic and metabolomic profiling reveals the regulatory mechanism underlying salt tolerance in maize

**DOI:** 10.1186/s13059-025-03766-5

**Published:** 2025-09-10

**Authors:** Fei Zhang, Boming Ji, Si Wu, Jie Zhang, Hui Zhang, Fei Wang, Baoxing Song, Qing Sang, Wenjie Huang, Shijuan Yan, Mustafa Bulut, Yariv Brotman, Mingqiu Dai

**Affiliations:** 1 National Key Laboratory of Crop Genetic Improvement, Huazhong Agricultural University, Wuhan, 430070 China; 2 Shandong Laboratory of Advanced Agriculture Sciences in Weifang, Peking University Institute of Advanced Agricultural Sciences, Weifang, 261325 China; 3 Department of Genetics, Stanford University, Stanford, CA USA; 4 Present Address: Precision Medicine Computational Biology, South San Francisco, Amgen, CA USA; 5 Key Laboratory of Maize Biology and Genetic Breeding in Arid Area of Northwest Region, College of Agronomy, Northwest A&F University, Yangling, Shaanxi 712100 China; 6 Guangdong Key Laboratory for Crop Germplasm Resources Preservation and Utilization, Agro-Biological Gene Research Center, Guangdong Academy of Agriculture Sciences, Guangzhou, 510640 China; 7 Max-Planck-Institute of Molecular Plant Physiology, Am Mühlenberg 1, Potsdam-Golm, 14476 Germany; 8 Program Center MetaCom, Leibniz Institute of Plant Biochemistry, Halle (Saale), 06120 Germany; 9 School of Plant Sciences and Food Security, Tel Aviv University, Tel Aviv, 69978 Israel

**Keywords:** Maize, Time series, Multi-omics, Salt stress

## Abstract

**Background:**

Soil salinization represents a critical global challenge to agricultural productivity, profoundly impacting crop yields and threatening food security. Plant salt-responsive is complex and dynamic, making it challenging to fully elucidate salt tolerance mechanism and leading to gaps in our understanding of how plants adapt to and mitigate salt stress.

**Results:**

Here, we conduct high-resolution time-series transcriptomic and metabolomic profiling of the extremely salt-tolerant maize inbred line, HLZY, and the salt-sensitive elite line, JI853. Utilizing advanced data mining techniques, we identify key factors underlying the divergence in salt tolerance between these two lines and discover a series of novel genes and metabolites essential for maize salt tolerance. Additionally, we develop an innovative decision algorithm that enabled the construction of a high-confidence gene regulatory network for important salt-responsive metabolites. Comprehensive genetic and molecular studies further reveal the pivotal role of a hub gene, *ZmGLN2*, in regulating metabolite biosynthesis and salt tolerance in maize.

**Conclusions:**

Our study provides the first high-resolution transcriptomic and metabolomic dataset for crop salt response, uncovering novel maize salt-responsive genes and metabolites. These findings demonstrate the effectiveness of high-resolution multi-omics in deciphering the mechanisms underlying complex crop traits. Furthermore, we develop a systematic analytical framework for mining time-series multi-omics data, which can be broadly applied to other species or traits.

**Supplementary Information:**

The online version contains supplementary material available at 10.1186/s13059-025-03766-5.

## Background

Excessive salt in soil acts as a major environmental stressor, inhibiting plant growth, development, and yield, posing a significant threat to crop production worldwide. It is estimated that the annual agricultural economic losses due to soil salinization globally amount to $27.3 billion [1]. Developing and promoting salt-tolerant crop cultivars is an effective strategy to unlock the agricultural production potential of salinized farmland [2]. Comprehending the mechanisms underlying plant responses to salinity and identifying genes that confer salt tolerance are pivotal for advancing salt-tolerant crop breeding.

Currently, the cloning of salt-tolerant genes in plants primarily relies on forward genetic methods such as mutant screening, linkage analysis, and genome-wide association studies (GWAS). In *Arabidopsis*, mutant screening based on root bending assays has identified several salt hypersensitive mutants, including *sos3*, *scabp8*, *sos2*, and *sos1*. These mutants are associated with the SOS (salt overly sensitive) pathway, which regulates plant Na^+^ exclusion [3, 4]. Furthermore, a calcium ion imaging-based screening assay identified the Na^+^ sensing-defective mutant, *moca1*, shedding light on the Na^+^ sensing mechanism in *Arabidopsis* [5]. Through map-based cloning, several members of the HKT transporter family have been identified in rice, wheat, and maize [6–10], highlighting the critical and conserved roles of the HKT family in plant salt response.

Maize, a major cereal crop, faces yield threats from salt stress. Given the substantial genetic diversity and rapid linkage disequilibrium decay in natural populations, GWAS is widely utilized to unravel the genetic basis of salt tolerance in maize. Using ion concentration under salt stress as an indicator, GWAS has successfully identified several important salt-responsive genes in maize, including *ZmCBL8*, *ZmHKT1;2*, *ZmHAK4*, and *ZmRR1* [7, 11–13], which are pivotal in maintaining ion homeostasis under salt stress. Other salt-responsive genes in maize, such as *SAG4*,* SAG6*,* ZmCS3*,* ZmUGT*,* ZmCYP709B2*,* ZmCLCg*, and *ZmPMP3*, have been identified through indicators such as survival rates, metabolite contents, and growth rates under salt stress [14–16]. While these studies have significantly enhanced our understanding of plant salt responses, they often focus on a specific plant stage, lacking a comprehensive overview of plant responses to salt stress across different developmental stages.

The plant salt response is intricate and dynamic, with growth-limited factors and responsive mechanisms varying during salt stress [17]. Researchers have previously observed continuous growth changes in *Arabidopsis* roots under salt stress, delineating four distinct response phases: a rapid decrease in root growth rate (Stop phase, ST) after salt application, a temporary growth arrest (Quiescence, QU), a gradual recovery of root growth (Recovery, RE), and the establishment of a new growth equilibrium under salt stress (Homeostasis phase, HO) [18]. Transcriptome analysis at different salt response stages has revealed that hormones such as ABA, GA, JA, and BR orchestrate growth in salt stress response [18]. This study highlights the intricate nature of the plant salt response and emphasizes the significance of time-series omics data in elucidating plant salt tolerance mechanisms.

Time series design proves to be a potent tool for unraveling complex traits. Through the analysis of time-series transcriptome data, researchers have identified key gene modules involved in senescence, defense, drought resistance, and heterosis formation in *Arabidopsis* [19–22]. Utilizing time-series metabolic and transcriptomic profiles, critical metabolic changes and gene modules associated with *Arabidopsis* drought response have been identified [20]. In crop studies, a temporal network analysis in *Brassica rapa* has identified early physiological and transcriptomic indicators of mild drought, underscoring the vital roles of non-structural carbohydrates and circadian-related genes in early drought responses [23]. While time-series multi-omics data are rich in information and value, data mining remains a challenge. Existing studies often lack sufficient integration between different omics datasets, and high-resolution time-series datasets of maize under salt stress are still scarce, impeding a comprehensive understanding of maize salt responses.

In a previous study, we identified a salt-tolerant maize inbred line, HLZY [14]. Here, we generated high-resolution time-series transcriptomic and metabolic datasets for HLZY and JI853, a salt-sensitive inbred line widely used in maize breeding in China, under salt-stressed and non-stressed conditions. By employing various data mining techniques, we elucidated the response of these two lines under salt stress, identifying novel metabolites and genes crucial for maize salt tolerance. Moreover, we developed a novel LASSO-based decision algorithm for constructing gene regulatory networks and identified hub genes, including *ZmGLN2*, involved in salt tolerance through metabolic regulation. Our study offers insights into maize salt responses and genes essential for salt-tolerant maize breeding, along with a systematic analytical framework for mining time-series multi-omics data, which could prove valuable for dissecting other complex agronomic traits.

## Results

### Phenotypic differences between two maize inbred lines under salt stress

**Fig. 1:**
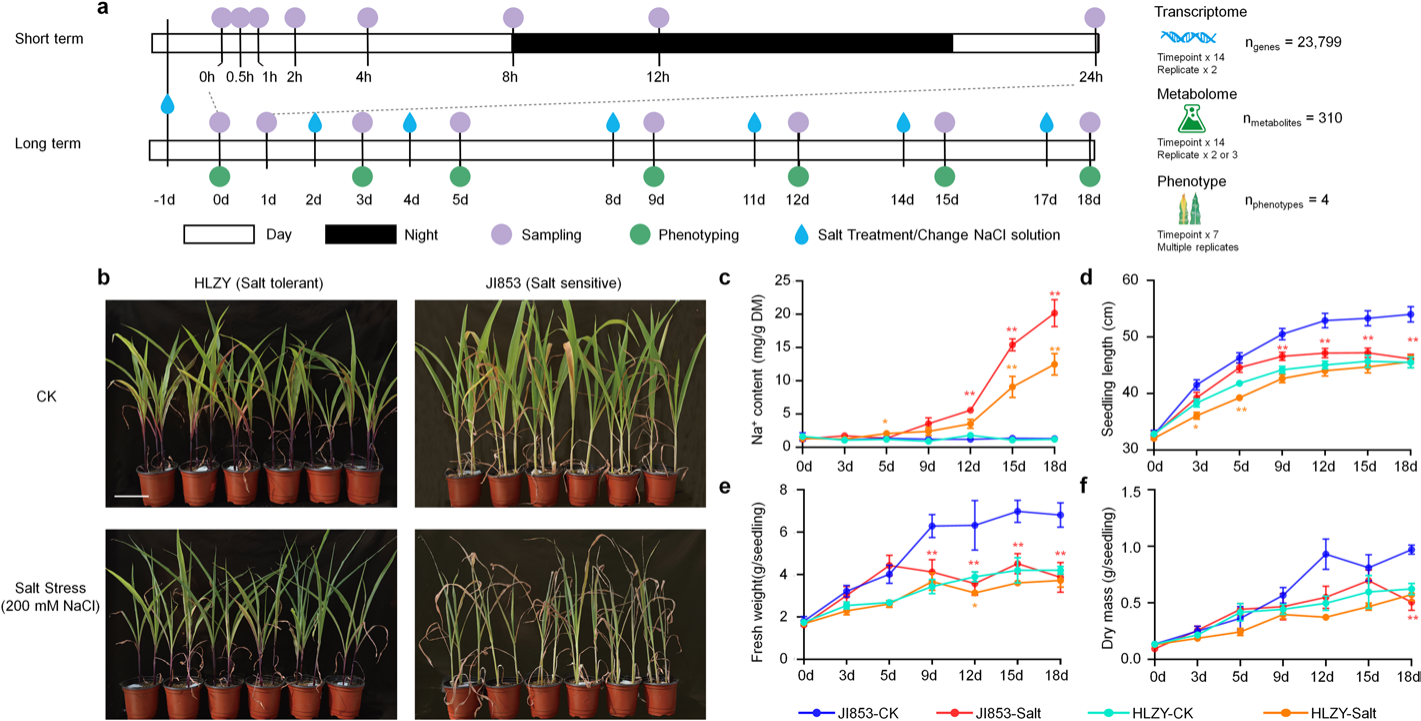
Experimental design and salt tolerance divergence of maize inbred lines HLZY and JI853. **a** Schematic illustration of the experimental design and the time point for sample harvest or phenotype investigation. **b** Visual comparison of HLZY and JI853 under control and salt-treatment conditions at 21 days after sowing (DAS). **c** HLZY, the salt-tolerant line, exhibits lower Na^+^ accumulation compared to the salt-sensitive line JI853. **d**–**f** Salt stress reduced seedling length (**d**), fresh weight (**e**), and dry mass (**f**) of two maize inbred lines at different stages. Scale bar, 10 cm. Error bar, standard deviation. Statistical significances in panel **c**–**f** were determined by group *t*-test, *P < 0.05, ** *P* < 0.01. Orange: HLZY-Salt vs HLZY-CK; red: JI853-Salt vs JI853-CK

In our previous study, we investigated the survival rates of a diverse collection of 445 maize accessions under salt stress conditions, identifying an exceptionally salt-tolerant inbred line (HLZY) and a salt-sensitive inbred line (JI853) [14]. While JI853 is a high-yielding elite line widely used in Chinese maize breeding [24]. To gain deeper insights into the dynamic responses of these two lines to salt stress, we subjected them to salt treatment and collected multiple omics datasets, including transcriptome and metabolome profiles, alongside various phenotypic responses, such as seedling length, fresh weights, dry mass, and shoot Na^+^ concentrations in a time-series manner (Fig. 1a). After 21 days of salt stress, all HLZY plants survived, whereas JI853 plants were near death (Fig. 1b).

In both lines, shoot Na^+^ levels began to increase on the 5th day after stress initiation (DAS). However, the rate of Na^+^ accumulation was notably lower in HLZY during the stress period (Fig. 1c, Additional file 2: Table S1), making the 5 DAS time point critical in our experiment. The growth inhibition observed before 5 DAS was likely due to osmotic stress from soil Na^+^, while growth inhibition post-5 DAS was primarily attributed to ionic stress resulting from intracellular Na^+^ accumulation.

Both HLZY and JI853 exhibited significant plant height inhibition shortly after exposure to salt stress (Fig. 1d). However, their response patterns differ across various stress stages. HLZY seedling lengths were more severely inhibited during the osmotic stress phase (0–5 DAS) compared to JI853. Conversely, no significant differences in plant height were observed between treatment and control groups during the ionic stress phase (5–18 DAS) for HLZY, whereas JI853 showed significant reductions during this phase (*P*-value at different time points is shown in Additional file 2: Table S1).

Salt stress leads to reductions in plant fresh weight and dry mass in both maize lines, but the timing of these reductions varied. In JI853, a significant decline in fresh weight relative to the control group was observed at 9 DAS (*P* = 0.026, Additional file 2: Table S1), whereas in HLZY, this decline occurred later at 12 DAS (*P* = 0.018, Additional file 2: Table S1). Regarding dry mass accumulation, HLZY experienced an early reduction in post-salt stress initiation, while in JI853, the reduction began during the ionic stress phase. Despite the earlier onset of dry mass reduction in HLZY, its dry mass continued to increase under salt stress throughout the experiment. In contrast, the dry mass of JI853 declined During the 15–18 DAS period (Fig. 1f).

### HLZY displayed a more global and rapid transcriptional response to salt stress compared to JI853

**Fig. 2:**
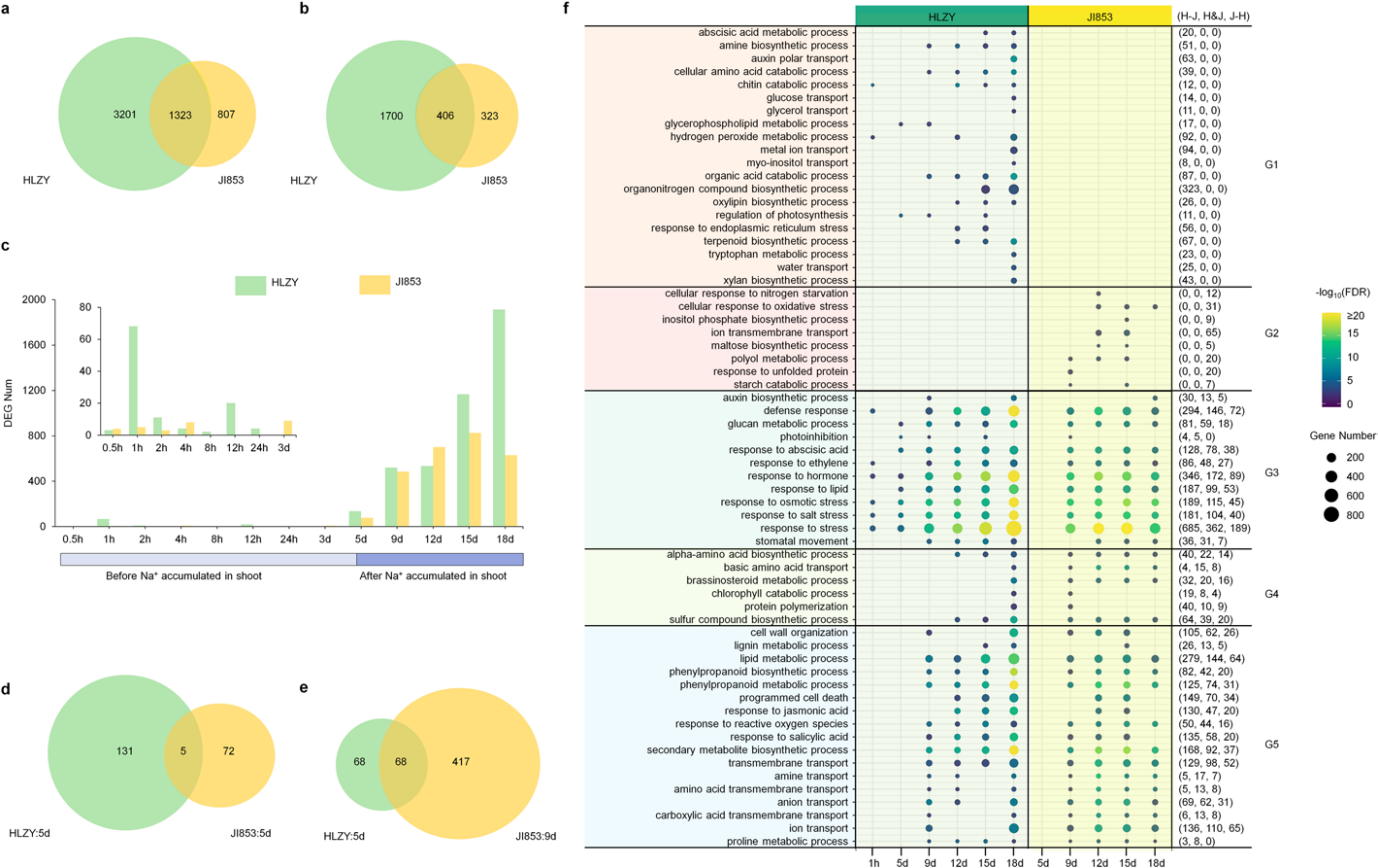
Transcriptional response to salt stress in HLZY and JI853. **a**–**b** Venn diagram of SSET genes (**a**) and DEAT genes (**b**) identified in HLZY and JI853. **c** Number of DEAT genes at each time point. **d** Venn diagram showing DEAT genes at 5 DAS in HLZY and JI853. **e** Venn diagram displaying DEAT genes at 5 DAS in HLZY and 9 DAS in JI853. **f** Chronology of reprehensive processes in HLZY and JI853 for salt response. The description of G1 to G5: G1, processes enriched specifically in HLZY; G2, processes enriched specifically in JI853; G3, processes enriched in both inbred lines, but with earlier enrichment occurrence in HLZY; G4, processes enriched in both lines, but with earlier enrichment in JI853; G5, processes firstly enriched at the same time in both lines, but involving different genes. H–J: associated genes specifically occurred in HLZY; H and J: associated genes occurred in both HLZY and JI853; J–H: associated genes specifically occurred in JI853

High-resolution time-series transcriptome profiling was performed to investigate the divergence in salt response at the gene expression level between HLZY and JI853 (Fig. 1a; Additional file 2: Table S2). In this study, we focused on genes showing salt-specific effects over time (SSET) in the two ecotypes, genes show salt-specific effect at one or more time points after time 0 are detected by a likelihood ratio test in HLZY and JI853 separately. We employed two formulas to model the gene expression: (1) a full formula that models the expression difference at time 0, the difference over time, and salt-specific differences over time; (2) a reduced formula that removes the salt-specific differences over time. Genes with salt-specific effects at one or more time points after time 0 will show smaller *P* values when comparing these two models (Additional file 1: Fig. S1, also see the “Methods” section). With a FDR (false discovery rate) threshold of < 0.01, we identified 4524 SSET genes in HLZY and 2130 in JI853, with only 1323 genes overlapping between the two lines (Fig. 2a, Additional file 2: Table S3). These results suggest that HLZY exhibited more extensive transcriptional changes in response to salt than JI853. To detect whether these SSET genes showed a significant salt effect at any specific time point, we applied a traditional Wald test to detect differentially expressed genes at each time point in HLZY and JI853 separately. These genes were determined as DEAT (differentially expressed at any time point) genes, with the threshold as FDR < 0.05 and |log2FC|≥ 1. Interestingly, only 45.60% (2106) of SSET genes in HLZY and 34.65% (729) in JI853 were detected as DEAT genes (Fig. 2b, Additional file 2: Table S4). SSET genes reflected the global transcriptomic salt response, while DEAT genes reflected the transcriptomic response at specific time points. The discrepancy between the number of SSET genes and DEAT genes indicates that many genes involved in the salt response had minor effects and exhibited cumulative changes over time.

From 0.5 h to 3 days after salt stress initiation, only a few of DEAT genes were detected. However, at 5 DAS, when Na^+^ accumulation in shoots increased, more DEAT genes were observed in both maize inbred lines (Fig. 2c). Notably, HLZY exhibited an earlier transcriptional response, while JI853 showed subtle transcriptional changes at this stage. Just 1 h after salt stress, 68 DEAT genes were identified in HLZY, enriched in pathways related to chitin metabolism, defense response, salt stress response, and hormone response pathways. At 5 DAS, dozens of DEAT genes were detected in both lines with minimal overlap (5 genes, 2.40%). On the contrary, DEAT genes at 5 DAS in HLZY showed more overlapping with those detected in JI853 at 9 DAS (68 genes, 12.3%. Figure 2d–e). This temporal shift suggests that HLZY mounts a quicker transcriptional response to salt stress compared to JI853.

### Chronology of salt response in two maize inbred lines

We examined the chronological responses of two maize inbred lines to salt stress to elucidate the mechanisms underlying their differing tolerances. Through Gene Ontology (GO) enrichment analysis of DEAT genes, we identified 627 unique enriched biological processes (FDR ≤ 0.01, gene count ≥ 5; Additional file 2: Table S5). These biological processes were categorized into five groups (G1–G5, see figure legend of Fig. 2) based on the timing differences in DEAT gene counts between the two inbred lines. Representative biological processes are illustrated in Fig. 2f, providing a detailed comparison of the transcriptional responses to salt stress in HLZY and JI853.

The salt tolerant inbred line HLZY exhibited an earlier response to primary salt stress and a slighter response to secondary salt-induced stresses. Elevated sodium concentrations in soil or plant tissues trigger osmotic and ionic stresses, leading to oxidative stress and subsequent secondary stress responses [25, 26]. Processes associated with primary stress responses, such as “response to stress,” “response to salt stress,” and “response to osmotic stress,” were enriched earlier in HLZY. In contrast, processes linked to secondary stress responses, like “cellular response to nitrogen starvation,” “cellular response to nitrogen starvation,” “cellular response to oxidative stress,” and “response to unfold protein,” were exclusively enriched in JI853. These findings suggest that the rapid primary stress response in HLZY may help mitigate or prevent secondary stress effects during salt exposure.

Phytohormones play a crucial role in plant response to salt stress by mediating signal perception, defense systems, growth regulation, and developmental adaptation [25]. Abscisic acid (ABA), ethylene (ET), salicylic acid (SA), and jasmonic acid (JA) exhibited differential timing and activity in the two lines [27]. Both HLZY and JI853 demonstrated sustained responses to ABA and ET signaling, but the initiation of these responses occurred earlier in the salt-tolerant line HLZY. Specifically, ABA response was first detected in HLZY at 5 DAS, whereas it was observed at 9 DAS in JI853. Similarly, ET response in HLZY occurred in two phases: an early transient phase at 1 h post salt stress and a sustained phase beginning at 9 DAS. In JI853, only the second phase of ET response was observed, coinciding with the timing in HLZY.

Differences in SA and JA responses were primarily observed in the duration and number of genes involved. While both lines initiated SA and JA responses simultaneously (9 DAS for SA and 12 DAS for JA), these responses ceased by 15 DAS in JI853 but persisted in HLZY until the end of the experiment. HLZY exhibited a significantly larger number of unique DEAT genes related to SA (193 genes) and JA (177 genes) to JI853 (78 SA-related and 67 JA-related DEAT genes), with 58 SA-related and 47 JA-related DEAT genes shared between the two lines.

Defense-related processes were enriched in HLZY as early as 1 h after salt stress onset, with genes like *ZmWRKY33* (GRMZM2G012724), *ZmERF1* (GRMZM2G123119), and several chitinases significantly upregulated in HLZY at this time point. Homologs of these genes in *Arabidopsis* are known to regulate plant defense and salt stress responses [28–30], suggesting that HLZY rapidly integrates salt stress signals with defense pathways.

Furthermore, processes related to metabolite metabolism and transport, photoinhibition, stomatal movements, cell wall organization, programmed cell death, and other physiological adaptations exhibited significant divergence between HLZY and JI853. These differences in biological processes likely underlie the variation in salt tolerance mechanisms between the two inbred lines.

### Temporal clustering identifies novel salt tolerance genes in maize

**Fig. 3:**
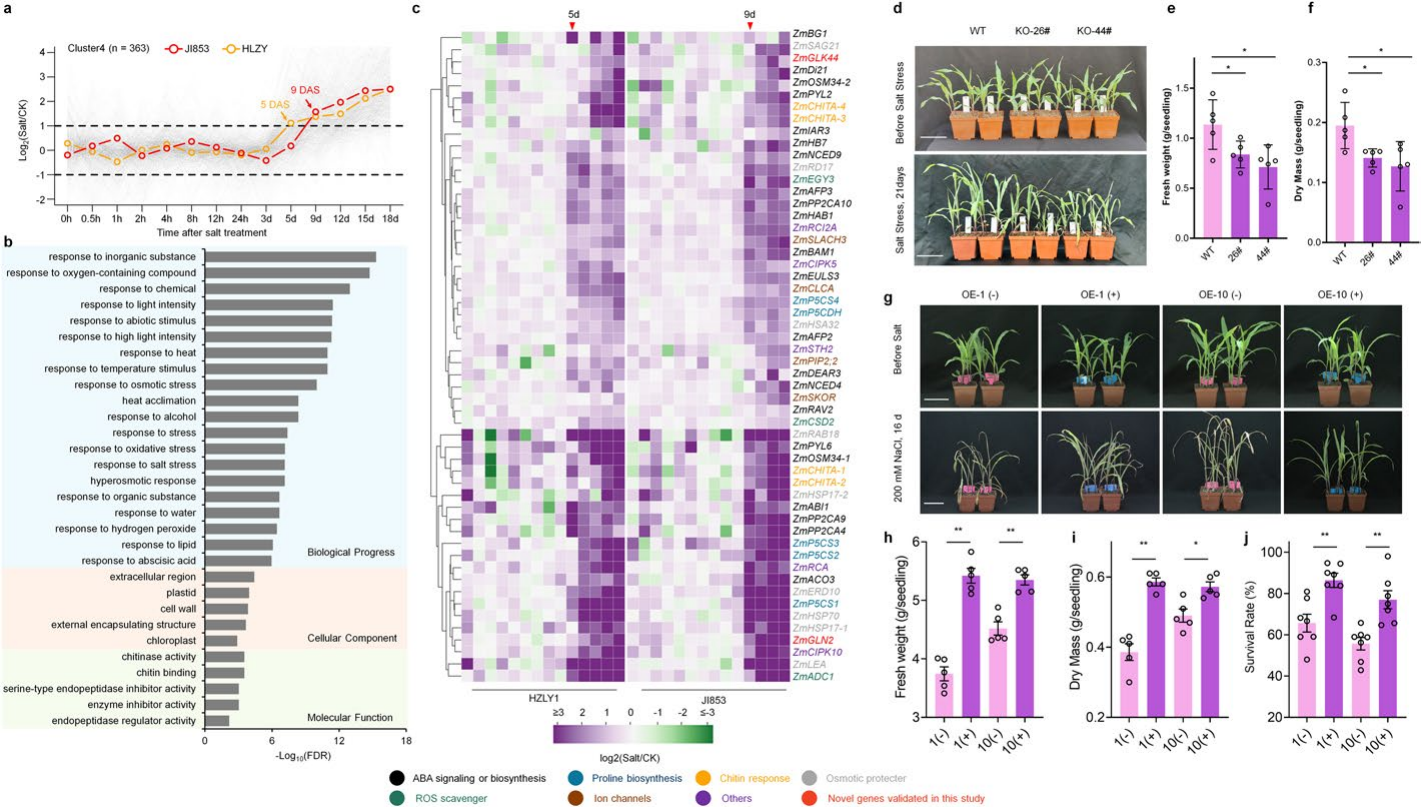
Clustering analysis identifies novel salt-tolerant genes in maize. **a** A total of 363 genes in Cluster 4 were activated earlier under salt stress in HLZY. **b** GO enrichment analysis of genes in Cluster 4. **c** Heatmap showing the expression patterns of known stress-responsive genes in Cluster 4. Gene functions are highlighted in different colors. **d**–**f** *ZmGLK44* knockout lines exhibited reduced salt tolerance compared to wild-type plants, with significantly lower fresh weight (**e**) and dry mass (**f**) under salt stress. **g**–**j** *ZmGLK44* overexpression lines displayed enhanced salt tolerance compared to negative control plants, showing improved fresh weight (**h**), dry mass (**i**), and survival rates (**j**) under salt stress. Scale bars: 10 cm. Statistical significance was determined by paired *t*-test; *P < 0.05, ** *P* < 0.01. Error bars: standard deviation

Transcriptomic and chronological analyses have revealed that HLZY exhibits a quicker transcriptional response to salt stress compared to the sensitive line JI853 (Fig. 2c, f). This accelerated response likely plays a key role in the stronger salt tolerance observed in HLZY. To identify genes with more rapid activation in HLZY, a temporal clustering analysis was performed on 5331 SSET genes based on the ratio of gene expression under salt stress relative to control conditions. This analysis categorized the genes into eight distinct clusters (Cluster 1 to Cluster 8; Additional file 1: Fig S2). Particularly, genes in Cluster 4 showed an interesting expression pattern with earlier salt-induced responses in HLZY. By 5 DAS, these genes displayed significant differential expression in HLZY, with an average fold change exceeding twofold compared to control samples. In contrast, the induction of these genes in JI853 was delayed, becoming evident only at 9 DAS (Fig. 3a). A total of 363 genes were comprised in Cluster 4 (Additional file 2: Table S6), predominantly associated with stress-responsive biological processes, as revealed by GO enrichment analysis. Cellular component enrichment indicated significant localization in the extracellular region, plastids, cell walls, and chloroplasts. Molecular function enrichment identified genes encoding proteins in Cluster 4 mainly involved in chitinase activity, chitin binding, and endopeptidase regulator activity (Fig. 3b).

Many genes in Cluster 4 have homologs implicated in salt stress responses in *Arabidopsis*. The expression pattern of these genes across the two inbred lines is shown in Fig. 3c. The function of these genes could be classified as ABA signaling or biosynthesis, proline biosynthesis, chitin response, osmotic protector, ROS scavenger, ion channels, and other stress-responsive (Fig. 3c, Additional file 2: Table S7). The significant concentration of known salt-responsive genes in Cluster 4 suggests that this group represents a consistent expression pattern among salt-tolerant genes in maize, raising a hypothesis that other uncharacterized genes within this cluster may serve as novel salt-responsive genes. To test our hypothesis, we selected two uncharacterized genes in Cluster 4, *ZmGLK44* and *ZmGLN2*, for further investigation.

*ZmGLK44* encodes a Golden 2-like transcription factor. Previously, its role in drought resistance was identified by overexpression transgenic studies [31]. To investigate its role in salt response, two independent CRISPR knockout lines of *ZmGLK44*, *KO-26#* and *KO-44#*, were generated. In *KO-26#*, a 306-bp sequence in the third exon was replaced by a 38-bp insertion, while *KO-44#* exhibited a 297-bp deletion (Additional file 1: Fig. S3). Under normal conditions, both knockout lines showed growth comparable to wild-type plants. However, under salt stress, these mutants exhibited significantly impaired growth, with markedly reduced fresh and dry weights relative to wild-type plants (Fig. 3d–f). Transgenic lines induced by the pRD101 promoter (1# and 10#) developed in previous studies were utilized to validate the function of *ZmGLK44* in maize salt response [31]. qPCR validation confirmed that the *pRD101* promoter successfully induced the expression of *ZmGLK44* (Additional file 1: Fig. S3). The transgenic plants showed significantly higher fresh weight, dry mass, and survival rates than their negative siblings (Fig. 3g–j). We did not observe significant sodium (Na^+^) levels, potassium (K^+^) levels, and Na^+^/K^+^ ratio changes among KO, OE, and wild types (Additional file 1: Fig. S4), indicating that *ZmGLK44* acts as a positive regulator of maize salt tolerance in the osmotic response phase but is not involved in the ion stress response. Overall, these findings highlight the efficacy of temporal clustering in identifying novel salt-responsive genes.

### Salt responsive metabolites are different between HLZY and JI853

**Fig. 4:**
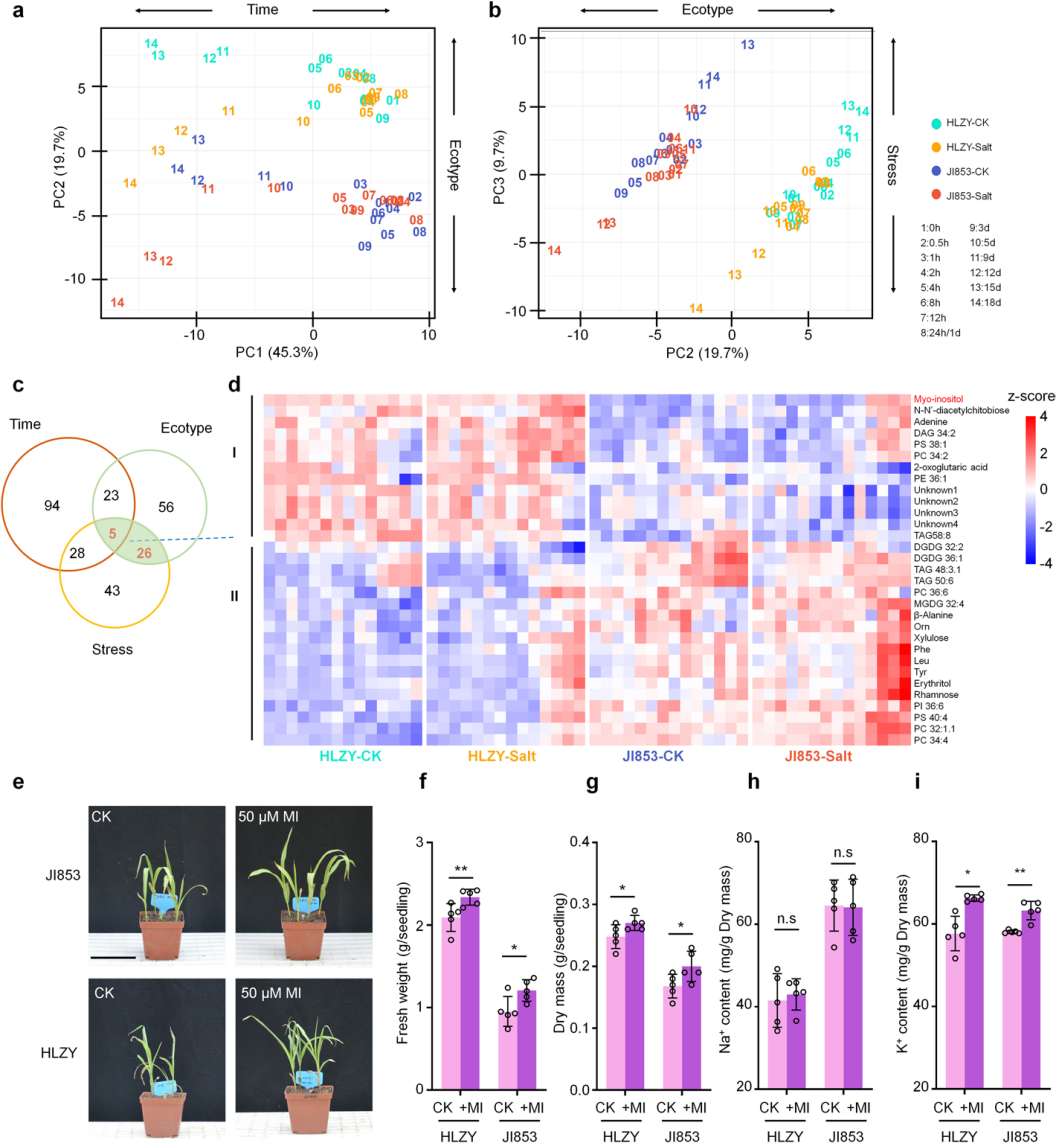
Metabolome response to salt stress and metabolic divergence between two maize inbred lines. **a**–**b** PCA of metabolic profiling. **c** Venn diagram displaying metabolites that significantly contribute to the PLS-DA model at the levels of “time,” “ecotype,” and “stress” with VIP scores above 1. **d** Heatmaps displaying the metabolic level of 31 metabolites significantly contribute to the classification in both “ecotype” and “stress” levels. Myo-Inositol (MI) is highlighted in red. **e**–**i** Application of exogenous MI enhanced salt performance of both lines (**e**). The level of fresh weight (**f**), dry mass (**g**), and shoot K^+^ content under salt stress were improved in the MI-treated group, while the shoot Na.^+^ content showed no significant change (**h**). Scale bars, 10 cm; Error bars: standard deviation; Statistical significance was determined by paired* t*-test, *P < 0.05, ** *P* < 0.01

Metabolic adjustments are essential for plant stress adaptation [17, 26, 32]. To explore the metabolic differences underlying salt tolerance in maize, we performed high-resolution metabolic profiling of the two inbred lines (Additional file 2: Table S8). Principal component analysis (PCA) of 125 primary metabolites and 185 lipids revealed distinct metabolomic changes. The first three principal components (PCs) explained 45.3%, 19.7%, and 9.7% of the variance, respectively. Notably, PC1 separated samples by time point, PC2 distinguished ecotypes, and PC3 grouped samples based on treatment (Fig. 4a, b).

Partial least squares discriminant analysis (PLS-DA) identified 31 metabolites as key contributors to samples classification in both “ecotype” and “stress” dimensions (VIP ≥ 1, Fig. 4c). These metabolites exhibited salt-induced patterns and baseline differences between HLZY and JI853, indicating their potential involvement in salt tolerance divergence. Among these, 13 metabolites, including myo-inositol, N’N-diacetylchitobiose, adenine, 2-oxoglutaric acid, DAG 34:2, PS 38:1, PC 34:2, PE 36:1, TAG 58:8, and four unidentified compounds, exhibited higher baseline levels in the salt-tolerant line HLZY. Conversely, 18 metabolites, such as β-alanine, ornithine, xylulose, phenylalanine, leucine, tyrosine, erythritol, rhamnose, and various lipid derivatives, were more abundant in the salt-sensitive line JI853 (Fig. 4c, Additional file 2: Table S8).

The changes in the metabolome closely corresponded with those observed in the transcriptome. For instance, proline content increased sharply between 5 and 9 DAS in JI853, whereas in HLZY, the rise in proline levels began earlier, between 3 and 5 DAS (Additional file 1: Fig. S4). This difference in proline accumulation aligns with the expression patterns of key enzymes in the proline biosynthesis pathway, such as *ZmP5CS1* (GRMZM2G028535). Additionally, N’N-diacetylchitobiose, a hydrolysis product of chitin, exhibited higher basal levels in HLZY. Chronological analysis revealed a specific enrichment of the “chitin catabolic process” in HLZY, while gene clustering analysis identified an earlier activation of chitinase-encoding genes in this line. In plants, the chitin response is closely linked to the salt stress response [13, 30, 33]. The elevated basal level of N′N-diacetylchitobiose in HLZY may indicate a more active chitin response, potentially contributing to its enhanced salt tolerance.

Myo-inositol, a critical metabolite involved in cell wall synthesis, intercellular signaling, and stress responses [34], was approximately 1.5 times more abundant in HLZY under non-stress conditions (Additional file 1: Fig. S4). The enzyme myo-inositol phosphate synthase (MIPS) regulated the rate-limited step in myo-inositol biosynthesis. Three salt-induced *MIPSs*—*ZmMIPS1-1* (GRMZM2G004528), Z*mMIPS2-1* (GRMZM2G392513), and *ZmMIPS2-2* (GRMZM2G177461)—were identified using a likelihood ratio test (FDR ≤ 0.01, also see the “Methods” section). *ZmMIPS1-1* and *ZmMIPS2-2* were expressed at a higher level in HLZY, whereas *ZmMIPS2-1* was more strongly expressed in JI853 (Additional file 1: Fig. S5). Exogenous application of myo-inositol enhanced biomass production and shoot K^+^ accumulation in both lines under salt stress without affecting shoot Na^+^ content (Fig. 4e–i). All these findings highlight the critical role of myo-inositol in salt stress tolerance and suggest that the higher baseline myo-inositol content in HLZY contributes to its superior salt tolerance.

### Constructing gene-metabolite regulated network using a novel LASSO-based decision algorithm

**Fig. 5:**
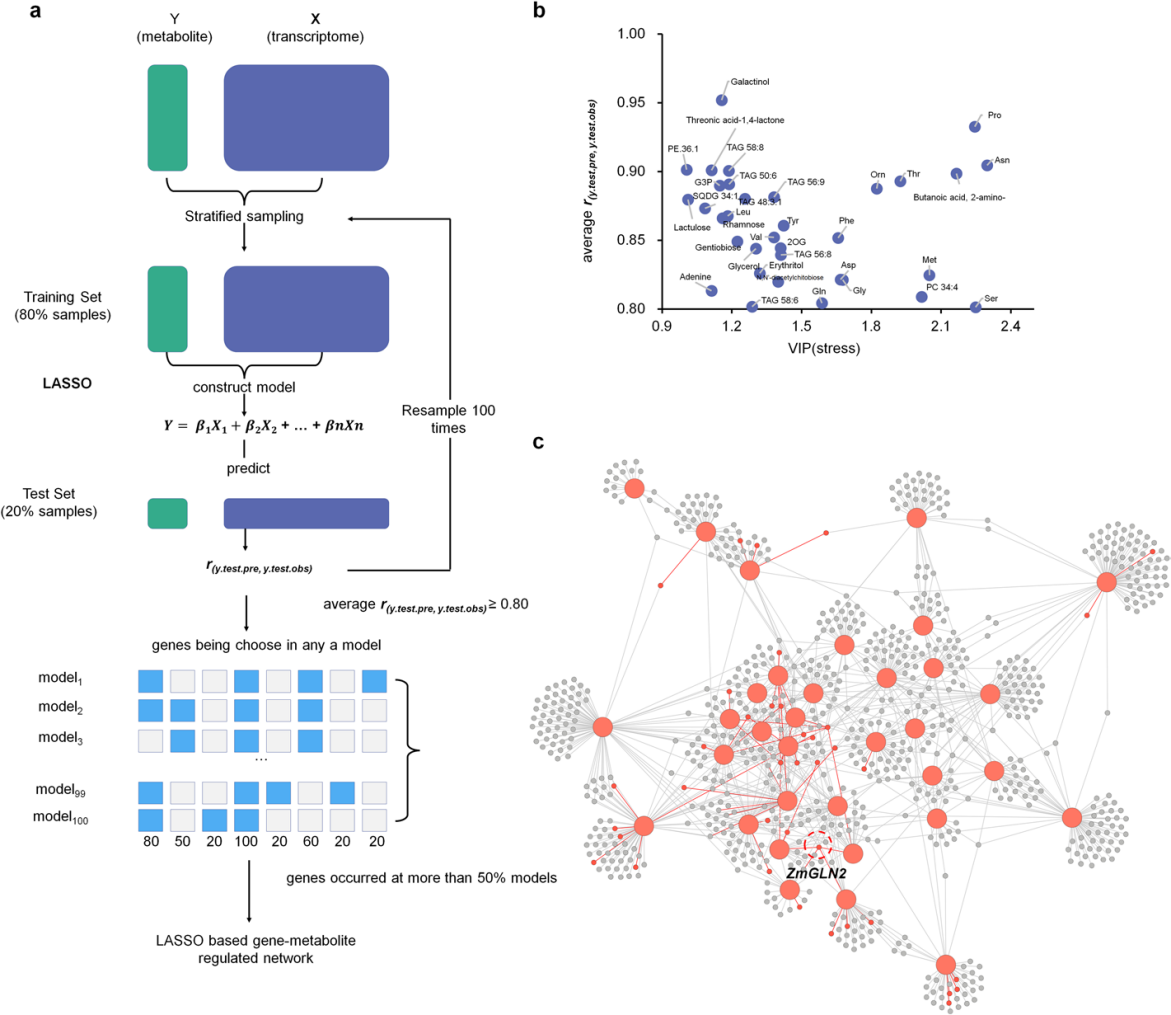
Construction of gene regulatory networks for key salt stress-responsive metabolites using a novel LASSO-based decision algorithm. **a** The workflow of the LASSO-based decision algorithm to select genes for predictable metabolites. **b** Prediction accuracy of 34 salt-responsive predictable metabolites. **c** Gene regulatory network of 34 predictable salt-responsive metabolites. Red circles represent metabolites, gray circles represent genes, and red edges indicate gene-metabolite interactions recorded in the STITCH 5 database

Metabolite abundances under specific conditions are influenced by the expression levels of related genes. However, regulatory genes for salt-responsive metabolites remain largely unidentified. To address this, we developed a LASSO-based decision algorithm to predict genes that influence specific metabolite levels. The schematic of this algorithm is illustrated in Fig. 5a. To improve model stability and reduce computational effort, Pearson correlation coefficients (PCC) were computed between all expressed genes and metabolites. For an individual metabolite, we retained only genes with PCC ≥ 0.5 as candidate variables for LASSO selection.

For each metabolite to be predicted, the samples were randomly assigned to a training set (80% of total samples) or a test set (20% of total samples). A LASSO model was applied to the training set to derive a multivariate linear regression model, which was then used to predict metabolite levels in the test set. The PCC between predicted and observed metabolite level, *r*_*(y.test.pre, y.test.obs)*_, was calculated to examine the prediction precision. This process was repeated 100 times, generating 100 prediction models and 100 *r* values. Metabolites with an average *r* value > 0.8 were classified as “predictable metabolites,” indicating that their levels could be determined by the expression of a set of genes. If a gene regulates a specific metabolite, it is expected to be repeatedly selected across the 100 models. Genes appearing in more than 50% of the models were considered as key regulators of target metabolites.

Out of 310 detected metabolites, models of 280 metabolites were successfully converged, identifying 90 predictable metabolites. Among these, 34 metabolites were classified as salt-responsive (VIP_stress_ > 1, Fig. 5b, Additional file 2: Table S9, Additional file 1: Fig. S6). Using these predictions, we constructed a gene-metabolite regulatory network comprising 34 salt-responsive metabolite nodes, 779 gene nodes, and 1200 edges representing predicted gene-metabolite associations. By cross-referencing with the STITCH 5 protein-compound interaction databases [35], we documented 50 gene-metabolite regulatory pairs in our network (Fig. 5c, Additional file 2: Table S9). Genes linked with 5 or more metabolites were identified as hub genes in this network.

### ZmGLN2 regulates amino acid accumulation in maize under salt stress

**Fig. 6:**
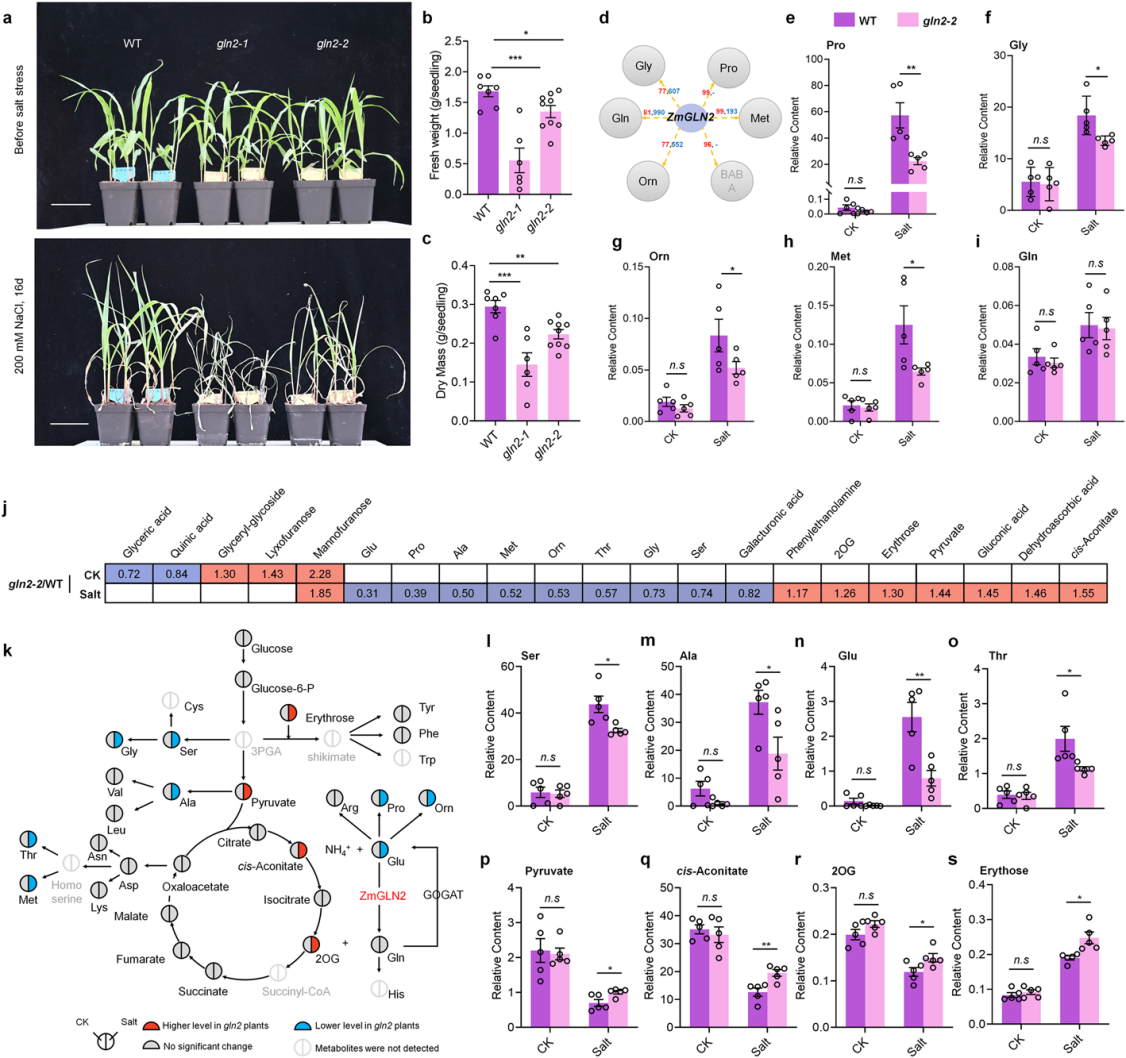
ZmGLN2 influences amino acid metabolism under salt stress in maize. **a**–**c**. Mutant of *ZmGLN2* showed a reduced salt tolerance (**a**), showing a decline of fresh weight (**b**) and dry mass (**c**) accumulated under salt stress. **d** Predicted regulation of Gly, Gln, Orn, BABA, Met, and Pro by *ZmGLN2*. The frequency of *ZmGLN2* in 100 prediction models for each metabolite is marked in red, and STITCH 5 scores are marked in blue. Metabolite BABA, not detected in *ZmGLN2* mutant *gln2-2* via GC–MS, is labeled in gray. **e**–**i** Pro, Gly, Met, Orn, and Gln level changes in *gln2-2* mutant and wild type plants under control and salt conditions. **j** Significantly changed metabolites between the *gln2-2* mutant and wild-type plants under CK and salt conditions. **k** Schematic of maize amino acid metabolic network, highlighting relative metabolite levels in the *gln2-2* mutant and wild-type plants under CK and salt conditions. **l**–**o** significantly reduced amino acid levels (Ser, Ala, Glu, Thr) in the *gln2-2* mutant under salt stress. Amino acids Pro, Met, Orn, and Gly are already shown in Fig. 7. **p**–**s** Elevated levels of carbon skeleton-providing metabolites (Pyruvate, *cis*-Aconitate, 2OG, and Erythrose) in the *gln2-2* mutant under salt stress. Statistical significance was determined by paired *t*-test, *P < 0.05, ** *P* < 0.01. Error bars, standard deviation

The temporal clustering and network analysis have both highlighted the significance of a particular gene, *ZmGLN2*, in maize salt response and metabolic regulations. *ZmGLN2* encodes a cytoplasmic glutamine synthetase (GLN/GS) that plays a central role in the GS/GOGAT cycle, responsible for 95% of nitrogen assimilation in plants. Among six GLN-encoding genes in the maize genome, only *ZmGLN2* exhibited a salt-induced expression pattern (Additional file 1: Fig. S7). We obtained two independent mutants, *gln2-1* and *gln2-2*, from the maize EMS mutant bank [36]. These mutants carry premature stop codons at Gln (855, *gln2-1*) and Trp (1239, *gln2-2*) sites, respectively (Additional file 1: Fig S8). Under normal conditions, the growth of *gln2* mutants was comparable to that of wild-type plants (Fig. 6a) but exhibited higher sodium levels (Additional file 1: Fig. S10). However, under salt stress, both mutants exhibited significantly reduced biomass accumulation and sodium levels compared to wild-type plants (Fig. 6a–c, Additional file 1: Fig. S10). A notably lower number of differentially expressed genes (DEGs) between CK and salt conditions compared to wild-type plants (FDR ≤ 0.05 and log2|Salt/CK|≥ 1). Salt stress led to transcriptomic divergence between *gln2* and wild-type plants, with the number of DEGs between *gln2* and wild-type increasing under salt stress. Together, all these results demonstrate the critical role of *ZmGLN2* in maize salt response.

Notably, *ZmGLN2* emerged as a hub gene in the predicted gene-metabolite regulatory network (Fig. 5c), aligning with our clustering analysis results. In the network, *ZmGLN2* was predicted to regulate the abundance of six amino acids: Gly, Gln, Orn, BABA, Met, and Pro. STITCH 5 confirmed interactions between the protein ZmGLN2 and Gly, Gln, Orn, and Met (Fig. 6d). To further validate our predictions, we quantified metabolite levels in the *gln2-2* mutant and wild-type plants at 8 DAS under normal and salt-stress conditions. Under normal conditions, no significant differences in predicted metabolite levels were observed between the *gln2-2* and wild-type plants. However, under salt stress, the *gln2-2* mutant exhibited significantly reduced accumulation of Pro, Gly, Orn, and Met, but not Gln (BABA was not detected in our GC–MS data, Fig. 6e–i). Proline, a critical stress protector in plants, showed markedly reduced levels in the *gln2-2* mutant, potentially contributing to the observed reduction in salt tolerance. Notably, the interaction between ZmGLN2 and proline was not reported in STITCH 5; genes involved in proline metabolism were down-regulated in gln2 mutants (Additional file 1: Fig. S12, Additional file 2: Table S10). All these results underscore the capability of our algorithm to uncover novel gene-metabolite regulatory relationships.

Using GC–MS, we identified 127 metabolites in both *gln2-2* mutants and wild-type plants (Additional file 2: Table S11). Under normal conditions, three metabolites were upregulated and two were downregulated in the *gln2-2* mutants compared to wild-type plants, while under salt stress, eight metabolites were significantly upregulated and nine downregulated (*P* < 0.05, Fig. 6j). Among the nine downregulated metabolites, eight were amino acids, indicating that *ZmGLN2* plays a key role in amino acid metabolism under salt stress (Fig. 6j).

Figure 6k summarizes changes in metabolites involved in amino acid synthesis under normal and salt stress conditions. While *ZmGLN2* deficiency did not affect amino acid metabolism under normal conditions, it significantly reduced the level of eight amino acids—Gly, Ser, Ala, Thr, Met, Pro, Orn, and Glu—under salt stress (Fig. 6e–i, l–o). Notably, metabolites contributing carbon skeletons for amino acid synthesis, such as pyruvate, cis-aconitate, 2-oxoglutarate (2OG), and erythrose, were significantly increased in *gln2-2* mutants compared to wild-type plants (Fig. 6k, p–s). This suggests that the *ZmGLN2* loss-of-function mutant disrupts the balance of carbon and nitrogen metabolism, leading to impaired amino acid biosynthesis under salt stress. Taken together, these findings, in conjunction with the clustering analysis (Fig. 3), demonstrate the essential role of *ZmGLN2* in maintaining amino acid levels under salt stress, highlighting its crucial involvement in regulating maize amino acid metabolism during salt adaptation.

## Discussion

Time series multi-omics data provides a dynamic insight into various traits, making it a powerful tool for unraveling complex processes like plant salt responses [18, 37]. However, these datasets are characterized by high dimensionality and substantial noise, posing significant challenges for data analysis and biological interpretation. In this study, we performed high-resolution time-series transcriptomic and metabolic profiling for the maize extreme salt-tolerant inbred line HLZY and the salt-sensitive elite line JI853 under both salt stress and control conditions. By employing a range of methods, including PLS-DA, likelihood ratio test, temporal clustering, chronological analysis, and network analysis, we achieved the following objectives: (1) delineated the transcriptional and metabolic differences in salt responses between HLZY and JI853; (2) identified and experimentally validated novel maize salt-responsive genes, such as *ZmGLK44* and *ZmGLN2*; and (3) constructed a high-confidence gene regulatory network for salt-responsive metabolites, elucidating a novel metabolic regulatory mechanism of the hub gene *ZmGLN2* under salt stress.

We propose a novel algorithm for constructing gene-metabolite regulatory networks. This algorithm utilizes LASSO [38] for variable selection, employing random grouping and repeated sampling, and identifies high-confidence regulatory genes for specific metabolites based on their frequency of occurrence in constructed models. Compared to traditional PCC-based network construction methods, our approach offers several advantages: (1) it utilizes multivariate regression models that consider the impact of multiple genes on the target metabolite; (2) it evaluates the average correlation between predicted and observed values to filter out metabolites whose content cannot be accurately explained by a group of gene expression, thereby reducing false positive rates; and (3) the selection of high-frequency genes enhances model reliability.

The metabolic function prediction and validation for the gene *ZmGLN2* highlights the efficacy of our algorithm. In *ZmGLN2* mutant plants, 80% of the predicted metabolites showed a significant decrease in accumulation compared to wild-type plants, except for glutamine (Gln) which showed no significant change. Notably, the lack of significant changes in glutamine levels does not indicate a model misprediction. *ZmGLN2* encodes a glutamine synthetase, catalyzing the conversion of glutamate to glutamine within the GS/GOGAT cycle. As a direct product of glutamine synthetase, glutamine acts as an intermediate and is rapidly utilized upon production [39, 40]. Therefore, we speculate that this rapid turnover of glutamine accounts for the observed lack of significant changes in its content in *ZmGLN2* mutant line.

In comparison to the salt-sensitive inbred line JI853, the salt-tolerant line HLZY exhibited broader and more rapid transcriptional responses to salt stress. HLZY displayed a significantly higher number of differentially expressed genes, particularly enriched in biological processes such as “chitin catabolism,” “defense,” “H_2_O_2_ metabolism,” “response to ethylene,” and “response to salt stress” within the initial hours of salt stress (even before Na^+^ accumulated in the shoot). As Na^+^ accumulation began in the shoot, genes related to ABA response, salt stress response, and photoinhibition were enriched in HLZY. In contrast, these processes were not enriched in JI853 until 9 DAS. By leveraging differences in transcriptional response rates, we identified and validated two novel salt-responsive genes, *ZmGLK44* and *ZmGLN2*. These findings highlight the critical role of expression dynamics in delineating salt tolerance disparities among different salt-tolerant maize lines. Furthermore, gene expression dynamics offer a potent avenue for discovering novel salt-responsive genes. The ability to characterize such dynamics hinges on high-resolution time-series experimental design, underscoring the unique advantages of time-resolved data in elucidating intricate biological processes like plant salt responses.

Metabolites such as myo-inositol and N,N′-diacetylchitobiose exhibited elevated basal levels in HLZY, potentially contributing to its enhanced salt tolerance. Exogenous application of myo-inositol or transgenic approaches to increase its endogenous levels in plants have demonstrated enhanced ROS scavenging capacity, mitigated oxidative stress induced by salt, and improved plant salt tolerance [41–43]. The ROS scavenging process mediated by myo-inositol interacts with ethylene and salicylic acid signaling pathways [41, 44], which are also significantly enriched in our transcriptomic data (Fig. 2f, Additional file 2: Table S5). N,N′-diacetylchitobiose, a disaccharide derived from the partial hydrolysis of chitin—a major component of fungal cell walls—is recognized by plants via the CERK1 receptor on the cell membrane [45]. Together with co-receptors such as LYKs and the calcium-dependent protein ANN1, CERK1 triggers the activation of immune responses [46]. Studies in *Arabidopsis* have demonstrated that the CERK1-mediated signaling pathway influences the salt stress response [33]. Our transcriptomic data further revealed differential expressions of genes encoding key enzymes involved in myo-inositol metabolism (MIPS) and chitinase, alongside enrichment of response processes associated with ethylene, salicylic acid, and chitin. Collectively, these differences in myo-inositol and chitin metabolism between HLZY and JI853 may underlie their different responses and tolerance mechanisms to salt stress.

In this study, we generated high-resolution time-series transcriptomic and metabolomic data for two maize inbred lines with contrasting salt tolerance capabilities, establishing a systematic multi-omics data analysis framework. We identified the underlying reasons for the differences in salt tolerance between the two lines at both the transcriptional and metabolic levels, constructed gene regulatory networks for key salt stress-responsive metabolites using a novel LASSO-based decision algorithm, and discovered and experimentally validated several novel salt-responsive genes. However, due to the incomplete genomic information available, this study was limited in fully exploring the natural variations between the two inbred lines. The availability of high-quality genome assemblies for HLZY and JI853, along with the development of recombinant inbred line (RIL) populations, would facilitate the systematic identification of natural variations that enhance salt tolerance in maize. This strategy promises to provide valuable resources for maize breeding programs and lays the foundation for future research endeavors.

## Conclusions

The salt stress response in plants is a highly dynamic process. At different stages of salt stress, the stress conditions encountered, the response mechanisms adopted, and the genes involved are varied. This presents a challenge for a comprehensive understanding of plant salt tolerance mechanisms [2]. Our study provides the first high-resolution transcriptomic and metabolomic dataset for crop salt response, uncovering novel maize salt-responsive genes and metabolites. By comparing with the chronology of two maize inbred lines in salt responding, our study significantly contributes to the understanding of maize responses to salt stress, offering theoretical insights for breeding salt-tolerant maize varieties and pinpointing new targets for genetic improvement. These findings demonstrate the effectiveness of high-resolution multi-omics in deciphering the mechanisms underlying complex traits. Moreover, we have developed an innovative analysis framework for multi-material, time-series, multi-omics datasets and proposed a novel data-mining algorithm, which can serve as a valuable reference for similarly designed studies.

## Methods

### Plant materials, growth conditions, and experimental design

Maize inbred lines HLZY and JI853 were sown and cultivated in pots (10 cm height, 8 cm diameter) filled with a standardized soil mixture (peat, domestic horticultural soil, and vermiculite at a 3:3:2 volume ratio). Ten pots were organized in a plastic tray (71cm × 40 cm × 15 cm). Six seeds were sown per pot, and three seedlings with consistent growth were retained after germination. These plants were grown in a greenhouse at Huazhong Agricultural University, Wuhan, China. Upon reaching the 1-leaf stage, salt treatment was initiated by adding 4 L of 200 mM NaCl to the trays at 9:00 a.m., while control trays received an equivalent volume of water. Samples for RNA sequencing and metabolomic analysis were collected at various time points on the first day of salt treatment (0 h, 0.5 h, 1 h, 2 h, 4 h, 8 h, 12 h, and 24 h). Additional samples were collected at 4, 5, 9, 12, 15, and 18 DAS (at 9:00 a.m., each time point). The NaCl solution and water in the trays were refreshed every 2–3 days.

Shoot samples from three randomly selected plants in HLZY and JI853 were pooled, snap-frozen in liquid nitrogen, and stored at −80°C for metabolite and RNA extraction. Shoots from another three randomly selected plants were harvested and weighed to get the fresh weight. Next, these samples were dried at 105 °C for 2 h, followed by drying at 65 °C until the weight stabilized, and then ground into powder using a mixer/mill (MM400, Restch, Germany) at 30 Hz for 1 min. Approximately 100 mg of shoot powder was weighed into 25-mL glass test tubes, digested in 10 mL of 1M HNO_3_ in a boiling water bath for 30 min, cooled, and filtered into 25-mL volumetric flasks with the volume adjusted using 1M HNO_3_. Na^+^ content was measured using a flame photometer (M410, Sherwood, UK). Plant height was measured at 0, 3, 5, 9, 12, 15, and 18 days after salt treatment.

For salt treatments of transgenic and mutant lines, plants were grown in square plastic pots (10cm × 10 cm × 10 cm) with four plants per pot and 24 pots per tray. Salt treatment was initiated at the 3-leaf stage. Fresh weight, dry mass, and ion content were performed as described above. Survival rate was investigated in the 21 st day after salt treatment.

### Metabolic profiling for primary metabolites and lipids

For each sample, 100 mg of homogenized fresh material was treated with 1 mL pre-cooled methanol/methyl-tert-butyl-ether (1:3, Sigma-Aldrich, USA) and shaken for 15 min at 4 °C. Afterwards, the homogenate was incubated in an ice-cooled ultra-sonication bath and was supplemented with 650 μL methanol/water (1:3 v/v). After vortexing and centrifugation for 5 min at 13,523 rcf and 4 °C, results in a two-phase separation. Five hundred microliters aliquot from the lower (semi-)polar phase and 700 μL aliquot from the upper organic phase were taken and dried under vacuum.

The (semi-)polar dried extract was derivatized as described in Lisec et al. [47]. In brief, extracts are treated with methoxyamine hydrochloride in pyridine and incubated while shaking for 2 h, followed by the addition of N-methyl-N-(trimethylsilyl)trifluoracetamide (MSTFA). After 30 min of shaking and a final centrifugation, the supernatant is transferred and injected into the GC–MS.

For the organic phase to analyze lipids, the dried extract is resuspended in acetonitrile:2-propanol (7:3 v/v). After centrifugation, the supernatant is injected into the LC–MS system. The detailed protocol for the machine settings and in-depth treatment of the dried extracts can be found in Bulut et al. [48, 49].

### RNA-seq

Total RNA was extracted using the TRIzol kit. RNA sequencing was conducted by Annoroad Gene Technology (Beijing, China) using a 150-bp paired end sequencing method. Clean reads were mapped to the maize reference transcriptome (v3.31) using “RSEM” for gene expression quantification [50]. Batch effects were corrected using the *ComBat()* function in R package “sva” [51].

### Statistical analysis of time-series metabolic and transcriptomic profiles

The signal intensity of each metabolite was log-transformed. Principal component analysis (PCA) was performed using the R package “pcaMethods” [52] to evaluate the impact of salt stress on the maize metabolome. Partial least squares discriminant analysis (PLS-DA) was conducted with the R package “ropls” [53]. The likelihood ratio test for SSET gene detection was performed using the R package “DESeq 2” [54]. A full model was designed to account for expression differences between replicates, baseline expression differences at time 0, changes over time, and salt-specific changes over time, using the formula:

$$Expression \sim batch + stress + time + stress:time$$

A reduced model excluding salt-specific changes over time was defined as:

$$Expression \sim batch + stress + time$$

Since all variables were treated as factors, the “*stress*” term in the formula represents the expression difference at time 0. Detection of DEAT genes was conducted following the standard analysis pipeline in “DESeq 2”.

### Exogenous myo-inositol

HLZY and JI853 plants were cultivated in pots and grown to the three-leaf stage under standard conditions as described previously. A 4-L solution containing 50 μM myo-inositol was added to the trays for the treatment group, while the mock group received an equal volume of water. The myo-inositol pretreatment was maintained for 12 h, after which the solution was replaced with 4 L 200 mM NaCl solution. Fresh weight, dry mass, and ion content were measured 10 days after the initiation of salt treatment.

### Enrichment analysis

Maize GO annotation files were downloaded from website agriGO (https://systemsbiology.cau.edu.cn/agriGOv2/) [55] and GO enrichment analysis was performed using TBtools [56].

### Temporal clustering

The expression ratio between salt-treated and control check (CK) samples was calculated using the mean log-transformed TPM values. Hierarchical clustering of expression ratios was performed using the R package “pheatmap” [57]. Clusters within the hierarchical clustering tree were truncated at a fixed height of 14 to delineate clusters.

### Plasmid construction and plant transformation

To generate *ZmGLK44* gene-edited transgenic lines using CRISPR-CAS9, two guide RNAs (*gRNA1* and *gRNA2*) were designed, both targeting the third exon of *ZmGLK44*. The *ZmU6* promoter, gRNAs, and the single guide RNA (sgRNA) scaffold were amplified by overlapping PCR with primer *gRNA1* and *gRNA2*, using the binary vector *pCPB-ZmUbi-hspCas9* [58] as the template. The resulting PCR fragments were sequenced and inserted into *pCPB-ZmUbi-hspCas9* through a recombination reaction (Vazyme, Nanjing, China), yielding the *pCPB-Cas9-gRNA1-gRNA2* construct. The construction of *ZmGLK44* overexpression lines was described previously [31]. Maize transformation was performed by tWIMI Biotechnology (Changzhou, China) using the receptor line KN5585. Primer sequences are provided in Additional file 2: Table S12.

### LASSO-based decision algorithm

The principle and process of the algorithm are described in detail in the text and Fig. 5a. LASSO was implemented using the *glmnet()* function from the R package “glmnet” [59]. The hyperparameter λ was estimated with the *cv.glmnet()* function, using the following parameters: *family* = *“gaussian” nfolds* = *10, intercept* = *FALSE, alpha* = *1, and lambda* = *seq(0.01, 1, 0.01).* The λ value selected as the best hyperparameter corresponds to the most regularized model where the cross-validated error is within one standard error of the minimum. The LASSO model with the optimal λ was then constructed using the *glmnet()* function, and genes whose coefficients are not zero were kept as candidate genes in this single LASSO model.

### GC–MS for *gln2* mutants

After 8 days of salt treatment, seedlings of gln2 and wild type plants were snap-frozen in liquid nitrogen and stored at − 80 °C for metabolite extraction. These samples pre-cooled in liquid nitrogen were ground using a Mixer/mill (MM400; Retsch, Germany) with a steel ball for 30 s at 30 Hz. 50 mg powder of each sample was extracted following the procedures described in a previous study [60]. The extract was centrifuged at 23,128 g for 10 min at 4 °C. The fixed volume of 200 μL of the polar phase was transferred into a pre-labeled 1.5 mL microcentrifuge tube. Then the samples were dried in a SpeedVac concentrator without heating. The dried 200 μL aliquots from the polar phase for primary metabolite profiling were derivatized with N-methyl-N-(trimethylsilyl) trifluoroacetamide as described previously [61] and further analyzed using GC–MS (7890A-5975C, Agilent, USA). 1 µL was taken from each sample and injected into GC–MS at 270 °C in a split mode (50: 1) with helium carrier gas (> 99.999% purity) flow set to 1 mL/min and separated by a DB-35MS UI (30 m × 0.25 mm, 0.25 µm) capillary column. The temperature was isothermal for 4 min at 90 °C, followed by an 8 °C increase per min ramp up to 205 °C, then held constant for 2 min, and finally ramped up at a rate of 15 °C per min to 310 °C and held constant for 2 min. The transfer line temperature was set to 300 °C, and the ion source temperature was set to 230 °C. The mass range analyzed was from m/z 85 to 700. The Agilent MassHunter Qualitative Analysis software version B.06.00 (Agilent Technologies, Palo Alto, CA, USA) and Agilent MassHunter Quantitative Analysis software version B.07.01 (Agilent Technologies, Palo Alto, CA, USA) were both used for GC–MS data analyses. NIST library and in-house database established using authentic standards were used together for metabolite identification.

## Supplementary Information

Additional file 1: Fig S1. The schematic of likelihood ratio test to detect SSET genes. Fig S2. Temporal clustering of 5331 SSET genes. Fig S3. Generation of *ZmGLK44* Crispr and transgenic line. Fig S4. The metabolic profiles of proline and myo-inositol. Fig S5. Temporal expression profile of MIPS-coding genes in HLZY and JI853 Fig S6. Prediction accuracy of all 310 detected metabolites. Fig S7. Phylogenic analysis and temporal expression profile of 6 GLN-coding genes in maize. Fig S8. Identification of *ZmGLN2* stop-gained mutant in maize EMS bank. Fig S9. Na^+^ levels, K^+^ levels, and Na^+^/K^+^ ratio change among *ZmGLK44 *OE, KO, and wild-type plants under CK and salt stress conditions. Fig S10. Na^+^ levels, K^+^ levels, and Na^+^/K^+^ ratio change between *gln2* mutants and wild-type plants under CK and salt stress conditions. Fig S11. The Venn diagram of DEGs between *gln2* mutant and wild-type plants under CK and salt conditions Fig S12. Expression levels of genes involved in proline metabolism in *gln2* mutant compared with wild-type plants in CK and salt conditions.

Additional file 2: Table S1. Phenotype divergence between HLZY and JI853 under salt. Table S2. Time-series expression profiles. Table S3. SSET genes identified by likelihood ratio test in HLZY and JI853. Table S4. DEAT genes identified by Wald-test in HLZY and JI853. Table S5. GO enrichment of DEAT genes at each time point in HLZY and JI853. Table S6. Ratio cluster of 5331 SSET genes. Table S7. Well-known stress responsive genes in Cluster 4. Table S8. Normalized intensities of 310 metabolites. Table S9. Gene-metabolite network constructed by LASSO-based decision algorithm. Table S10. RNA sequencing of *gln2* mutants and WT plants under CK and salt conditions. Table S11. Metabolic change in *gln2* mutant and WT plants under CK and salt conditions. Table S12. Primers in this study.

## Data Availability

The RNA-seq reads generated in this study have been deposited to the Genome Sequence Archive in BIG Data Center, Beijing Institute of Genomics, Chinese Academy Science, and are publicly accessible at https://ngdc.cncb.ac.cn/gsa/browse/CRA021765 [62]. The metabolic raw data reported in this paper have been deposited in the OMIX, Chian National Center for Bioinformation/Beijing Institute of Genomics, Chinese Academy of Sciences, and are accessible at https://ngdc.cncb.ac.cn/omix/release/OMIX011277 [63].
