# Supplementary material for: High-resolution time-series transcriptomic and metabolomic profiling reveals the regulatory mechanism underlying salt tolerance in maize
Source: Genome Biol. 2025 Sep 10;26:275. doi: 10.1186/s13059-025-03766-5 (PMC12421767; doi:10.1186/s13059-025-03766-5)
Supplement: Supplementary file 1 — Additional file 1: Fig S1. The schematic of likelihood ratio test to detect SSET genes. Fig S2. Temporal clustering of 5331 SSET genes. Fig S3. Generation of ZmGLK44 Crispr and transgenic line. Fig S4. The metabolic profiles of proline and myo-inositol. Fig S5. Temporal expression profile of MIPS-coding genes in HLZY and JI853 Fig S6. Prediction accuracy of all 310 detected metabolites. Fig S7. Phylogenic analysis and temporal expression profile of 6 GLN-coding genes in maize. Fig S8. Identification of ZmGLN2 stop-gained mutant in maize EMS bank. Fig S9. Na+ levels, K+ levels, and Na+/K+ ratio change among ZmGLK44 OE, KO, and wild-type plants under CK and salt stress conditions. Fig S10. Na+ levels, K+ levels, and Na+/K+ ratio change between gln2 mutants and wild-type plants under CK and salt stress conditions. Fig S11. The Venn diagram of DEGs between gln2 mutant and wild-type plants under CK and salt conditions Fig S12. Expression levels of genes involved in proline metabolism in gln2 mutant compared with wild-type plants in CK and salt conditions. [file 13059_2025_3766_MOESM1_ESM.pptx]

## Slide 1
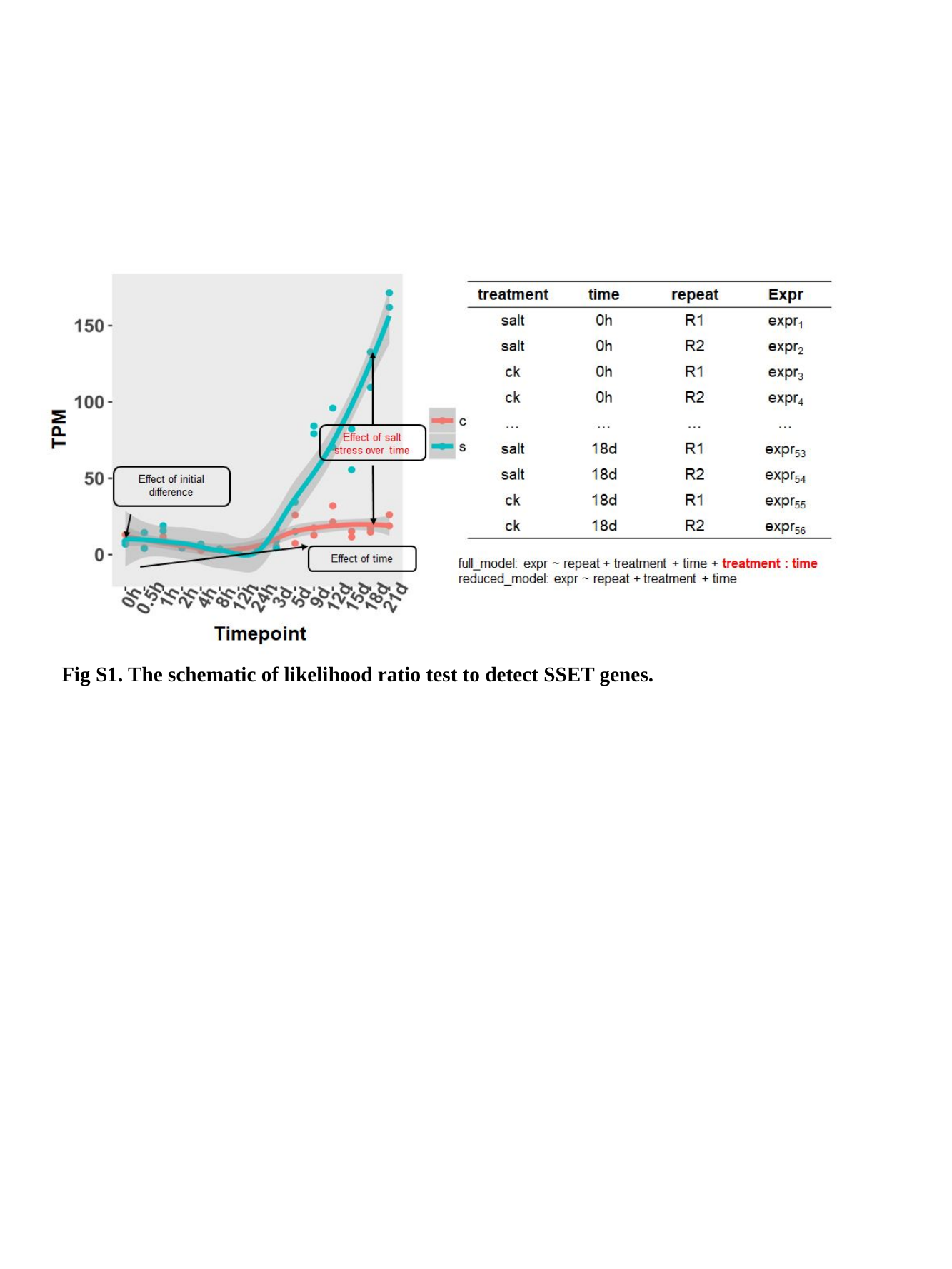

Fig S1. The schematic of likelihood ratio test to detect SSET genes.

## Slide 2
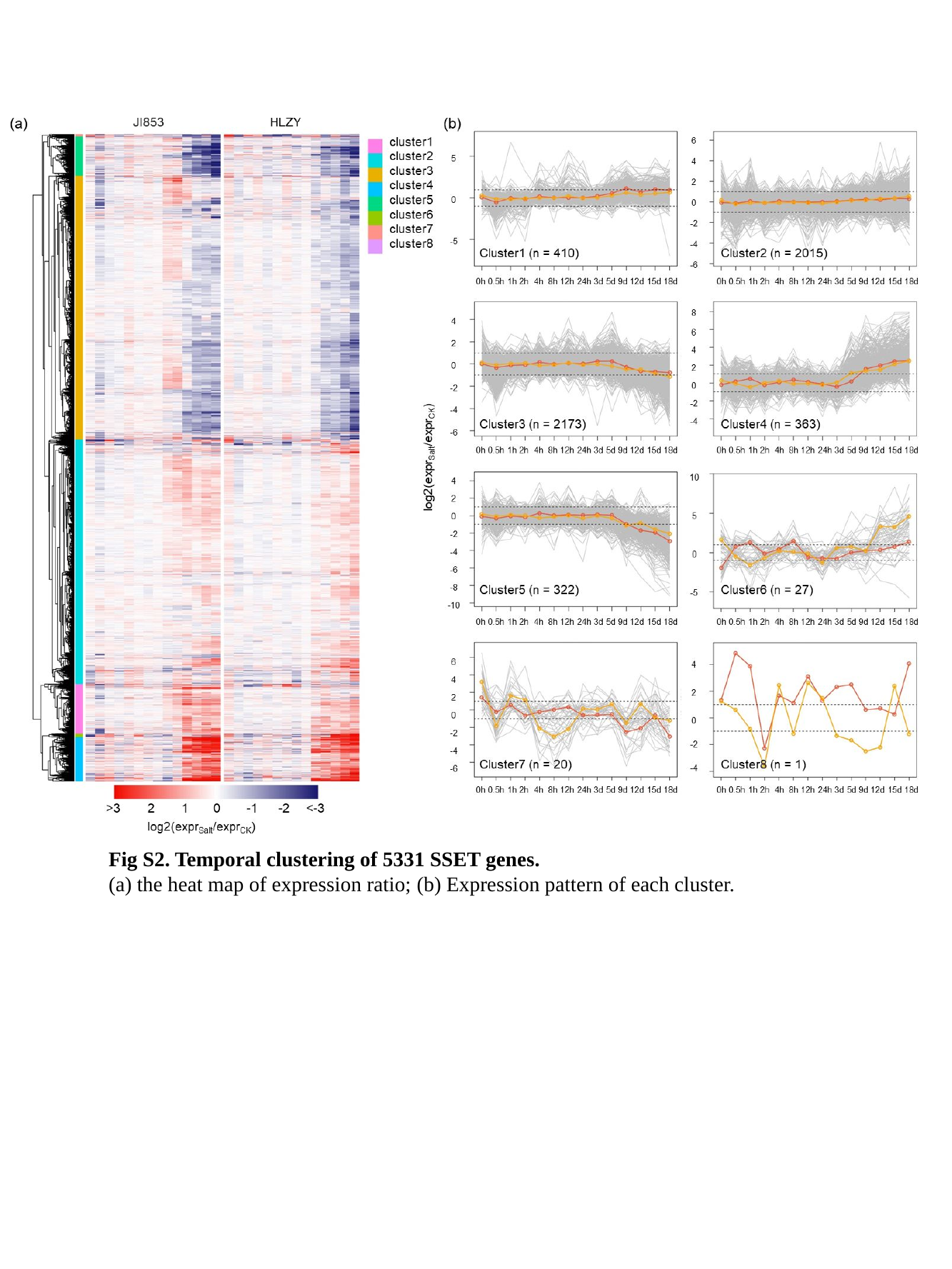

Fig S2. Temporal clustering of 5331 SSET genes.
(a) the heat map of expression ratio; (b) Expression pattern of each cluster.

## Slide 3
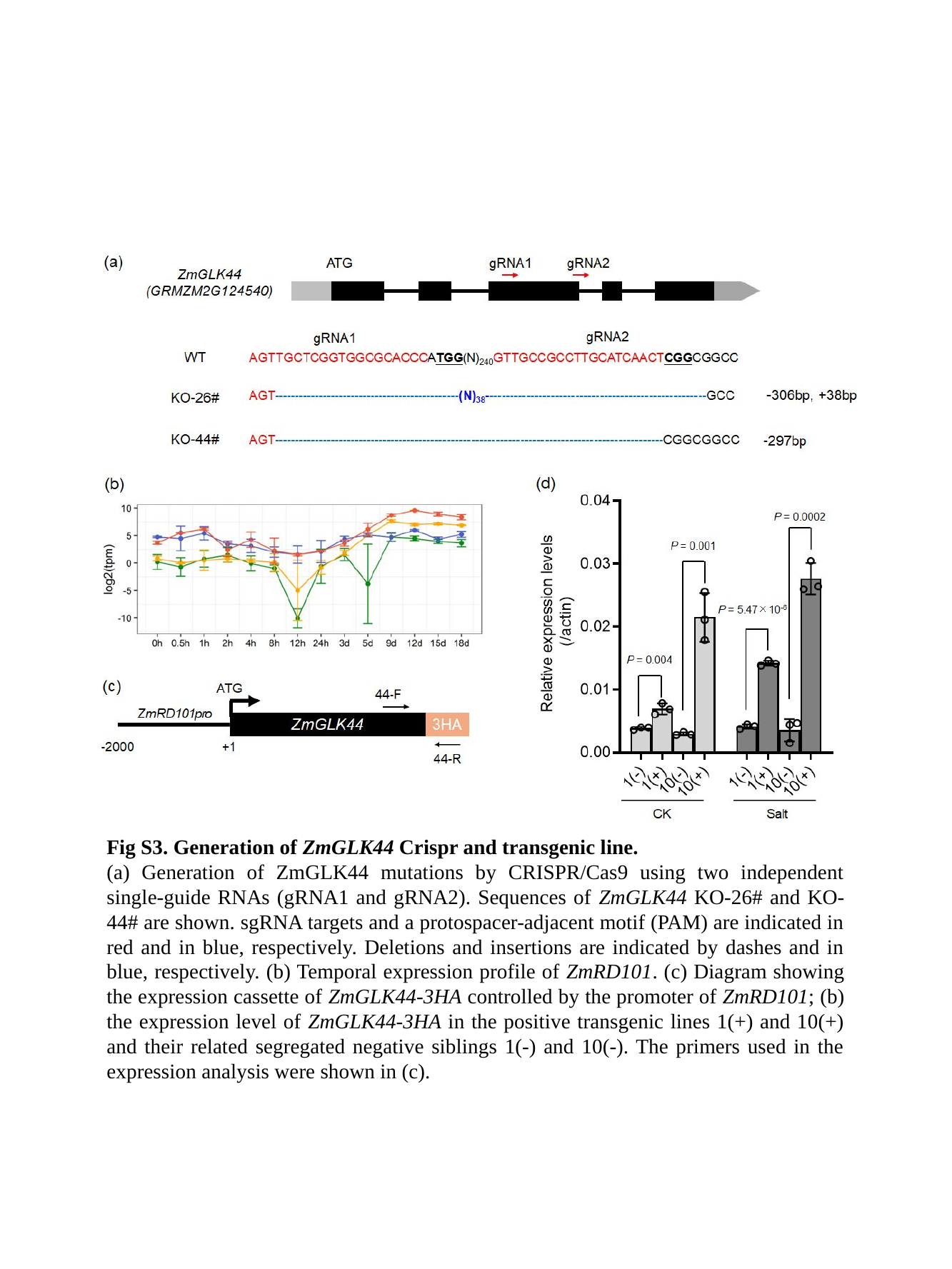

Fig S3. Generation of ZmGLK44 Crispr and transgenic line.
(a) Generation of ZmGLK44 mutations by CRISPR/Cas9 using two independent single-guide RNAs (gRNA1 and gRNA2). Sequences of ZmGLK44 KO-26# and KO-44# are shown. sgRNA targets and a protospacer-adjacent motif (PAM) are indicated in red and in blue, respectively. Deletions and insertions are indicated by dashes and in blue, respectively. (b) Temporal expression profile of ZmRD101. (c) Diagram showing the expression cassette of ZmGLK44-3HA controlled by the promoter of ZmRD101; (b) the expression level of ZmGLK44-3HA in the positive transgenic lines 1(+) and 10(+) and their related segregated negative siblings 1(-) and 10(-). The primers used in the expression analysis were shown in (c).

## Slide 4
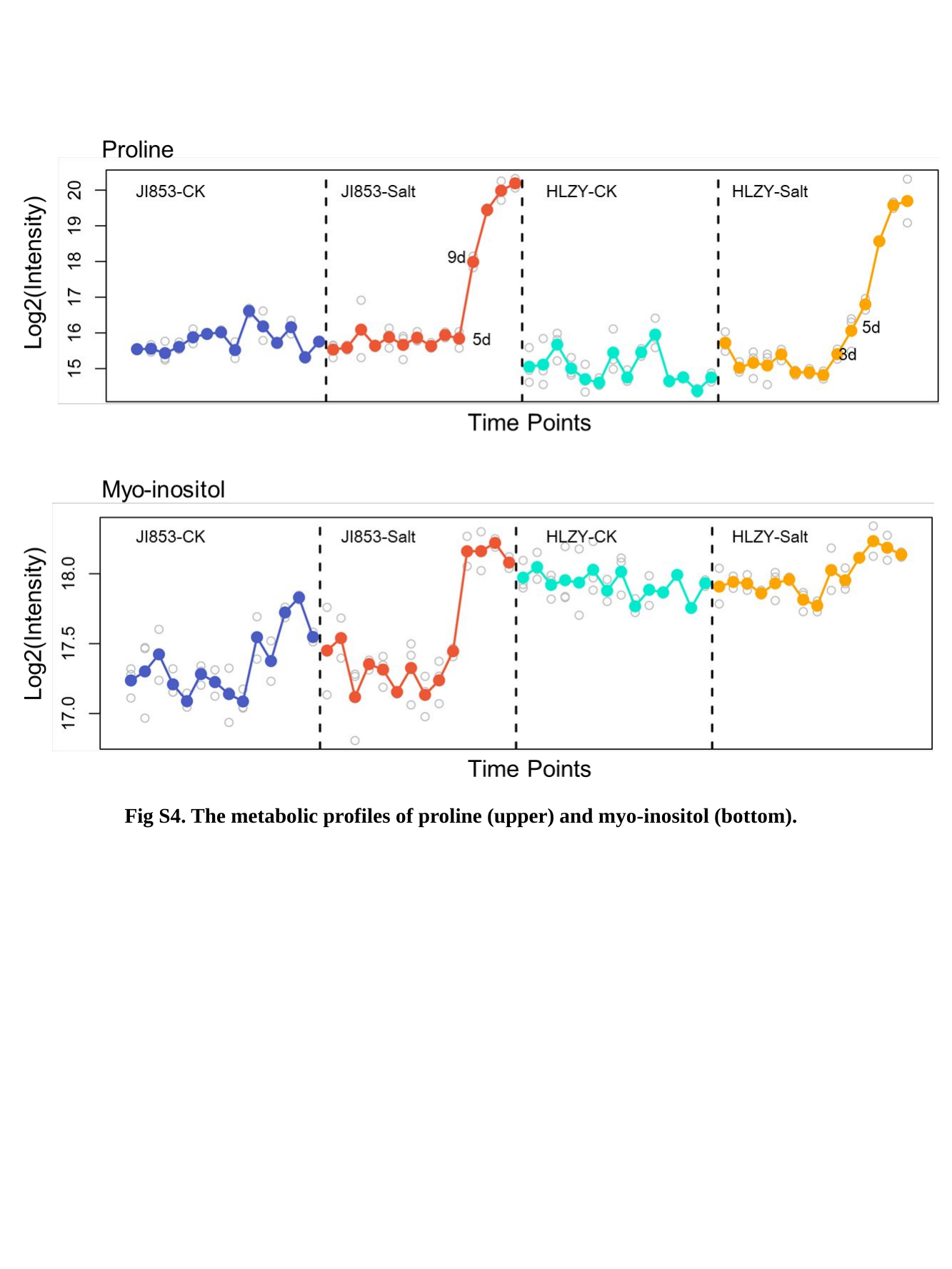

Fig S4. The metabolic profiles of proline (upper) and myo-inositol (bottom).

## Slide 5
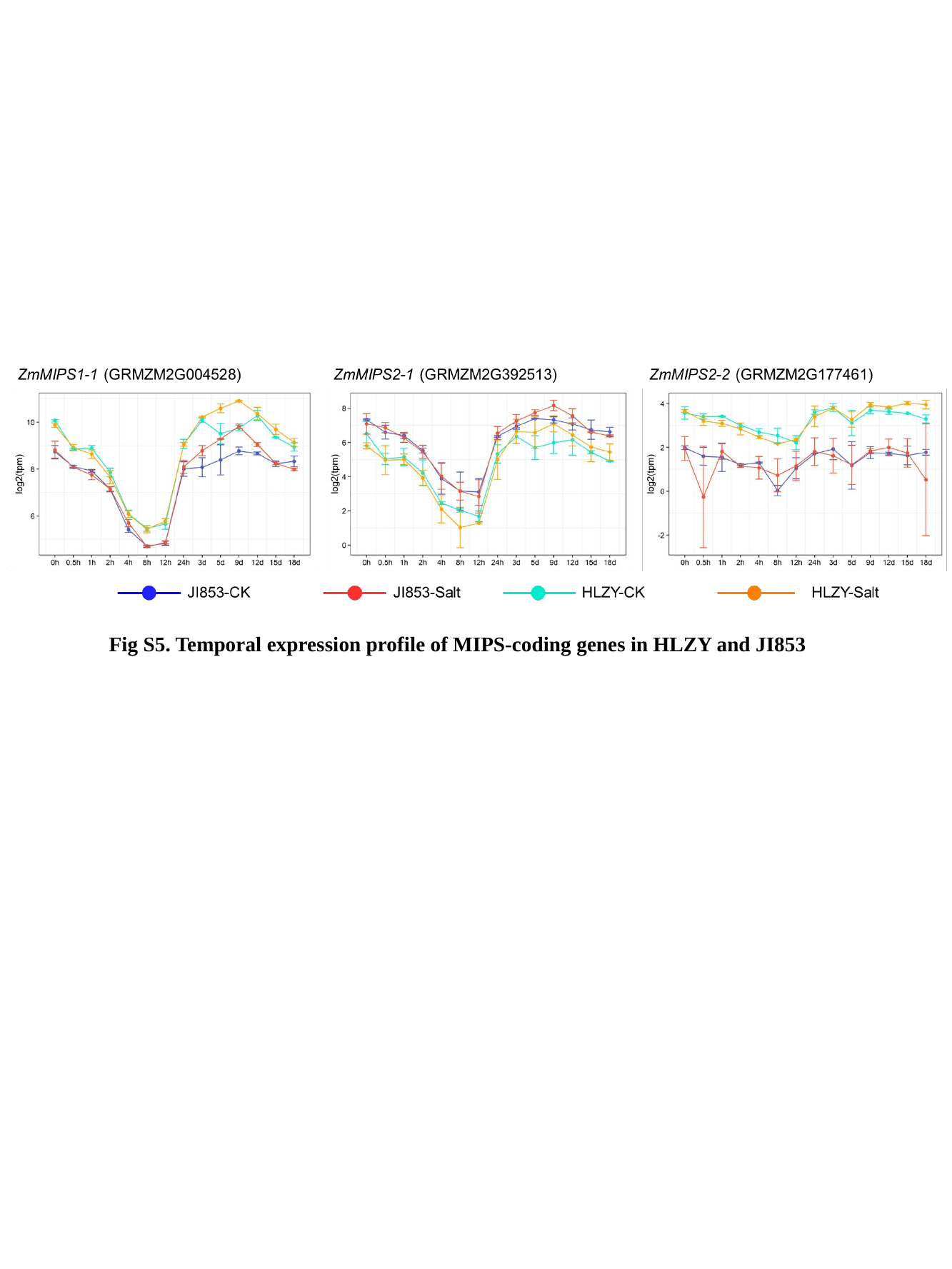

Fig S5. Temporal expression profile of MIPS-coding genes in HLZY and JI853

## Slide 6
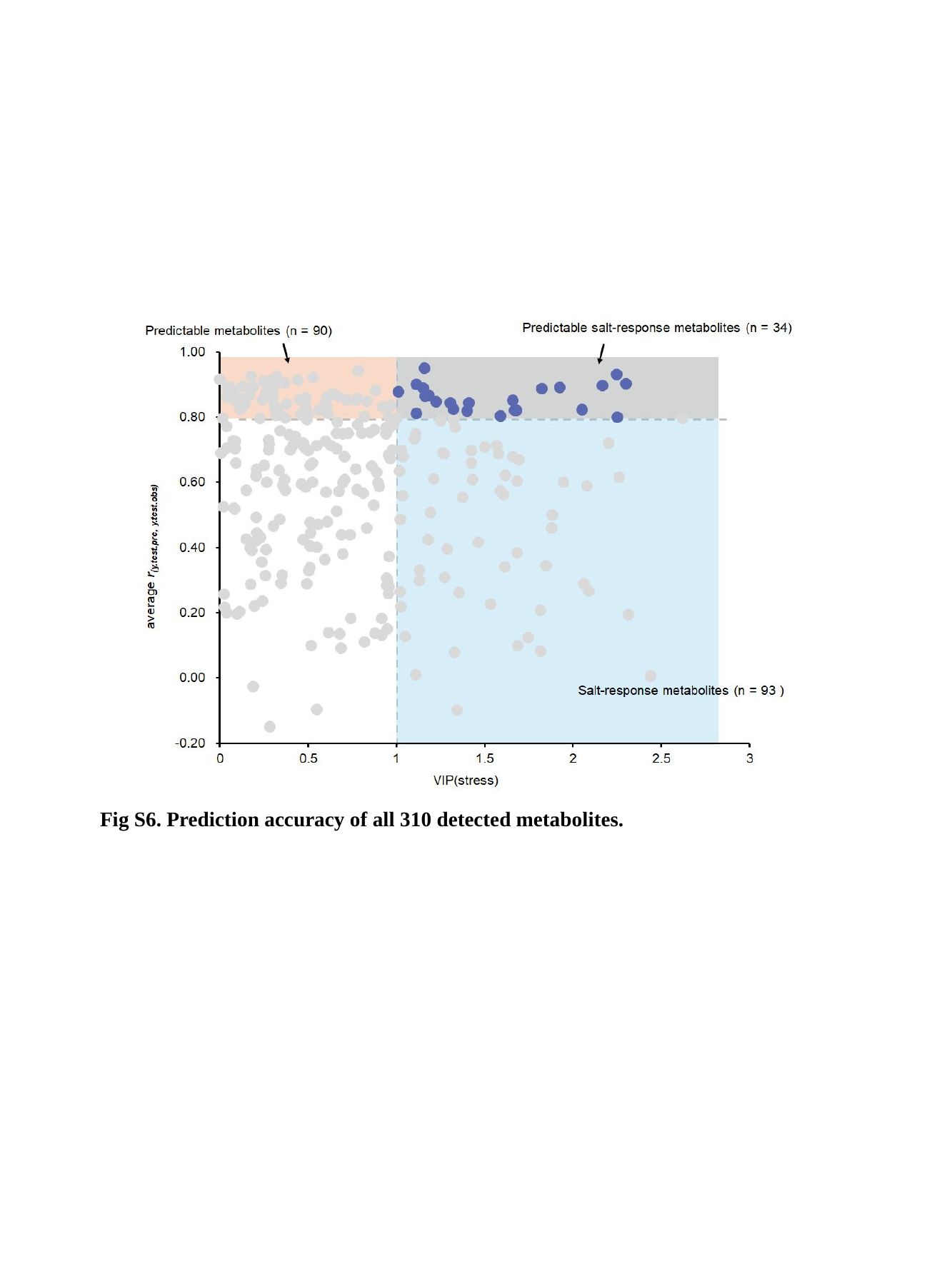

Fig S6. Prediction accuracy of all 310 detected metabolites.

## Slide 7
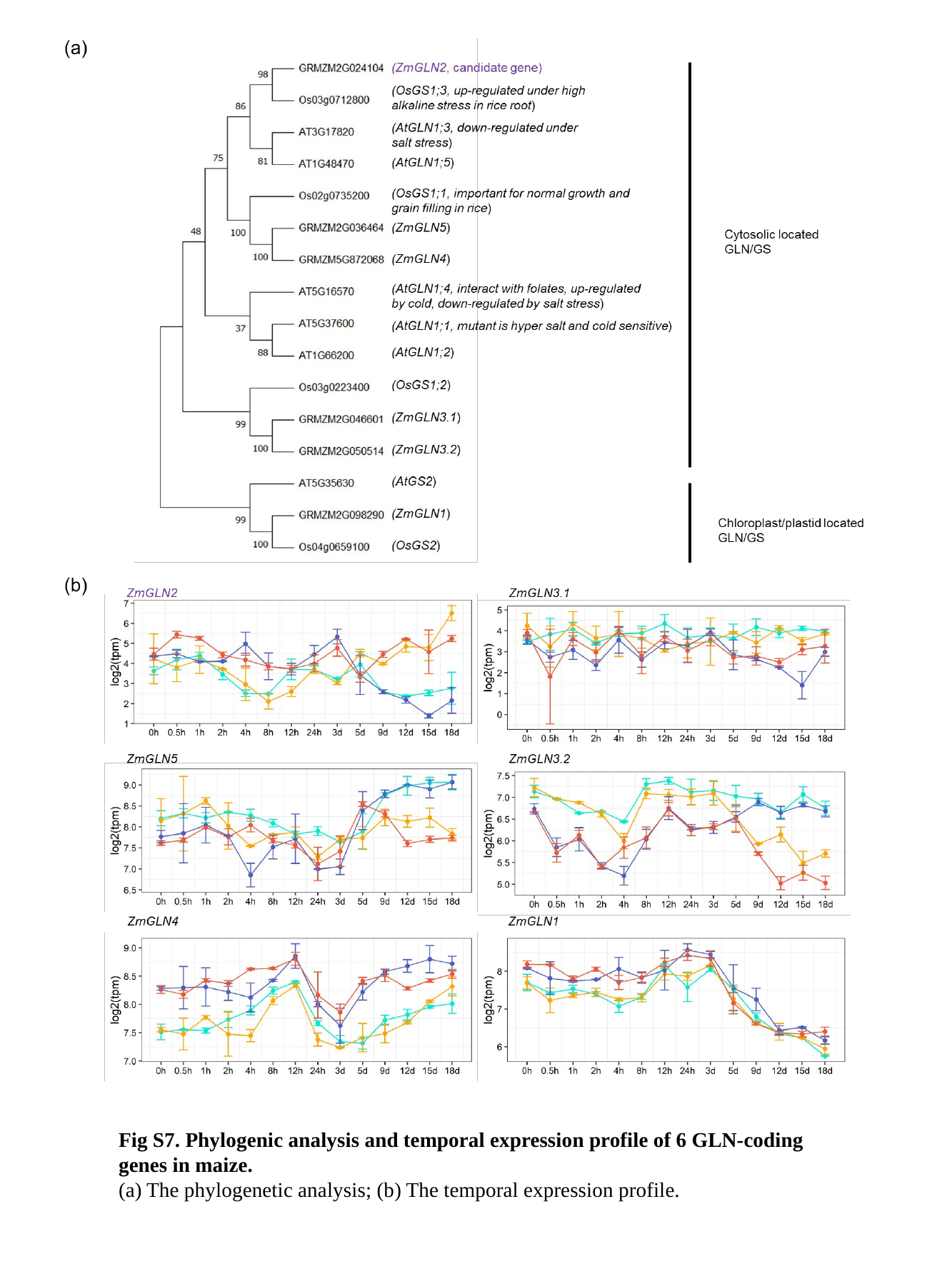

Fig S7. Phylogenic analysis and temporal expression profile of 6 GLN-coding genes in maize.
(a) The phylogenetic analysis; (b) The temporal expression profile.

## Slide 8
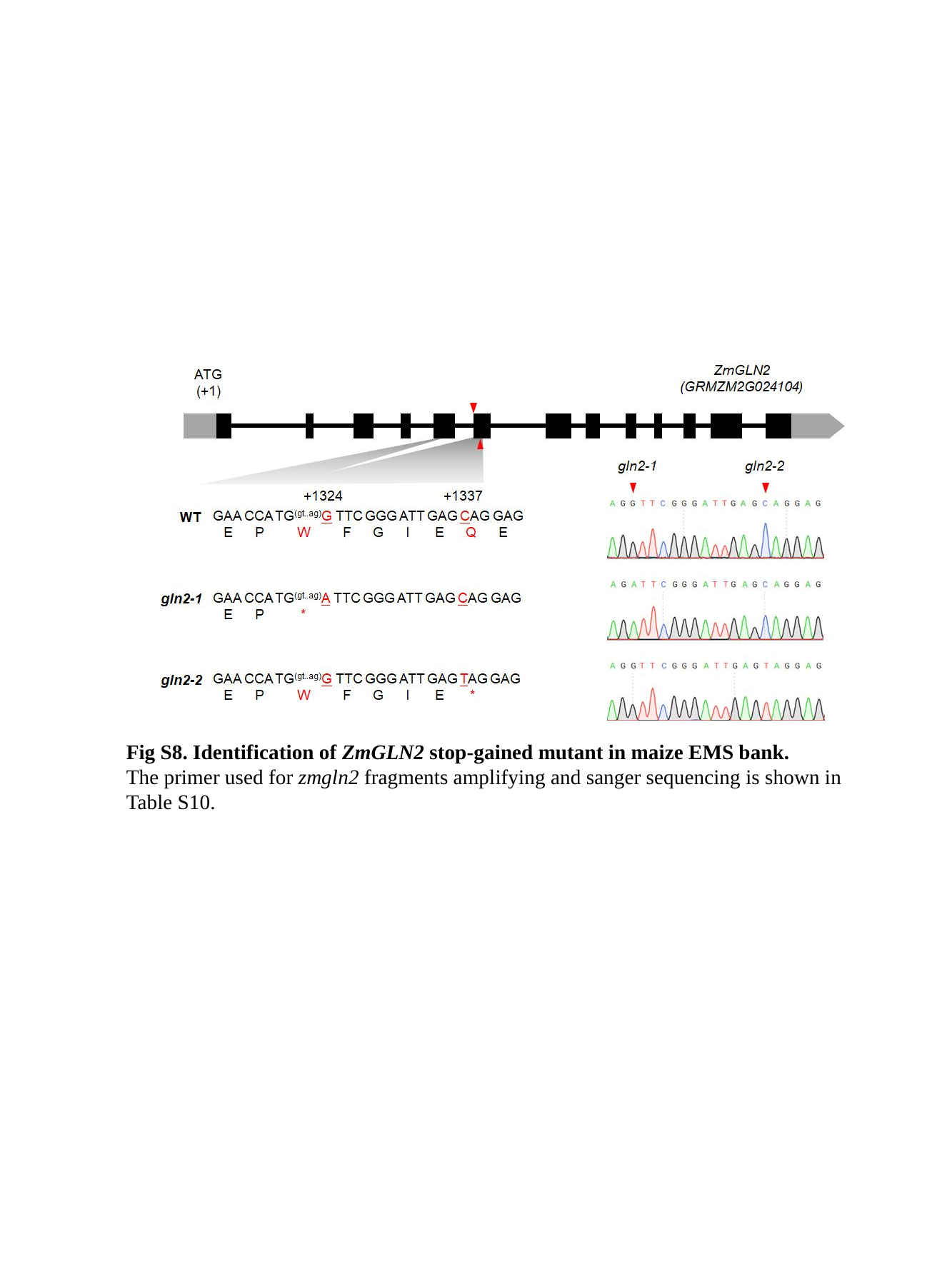

Fig S8. Identification of ZmGLN2 stop-gained mutant in maize EMS bank.
The primer used for zmgln2 fragments amplifying and sanger sequencing is shown in Table S10.

## Slide 9
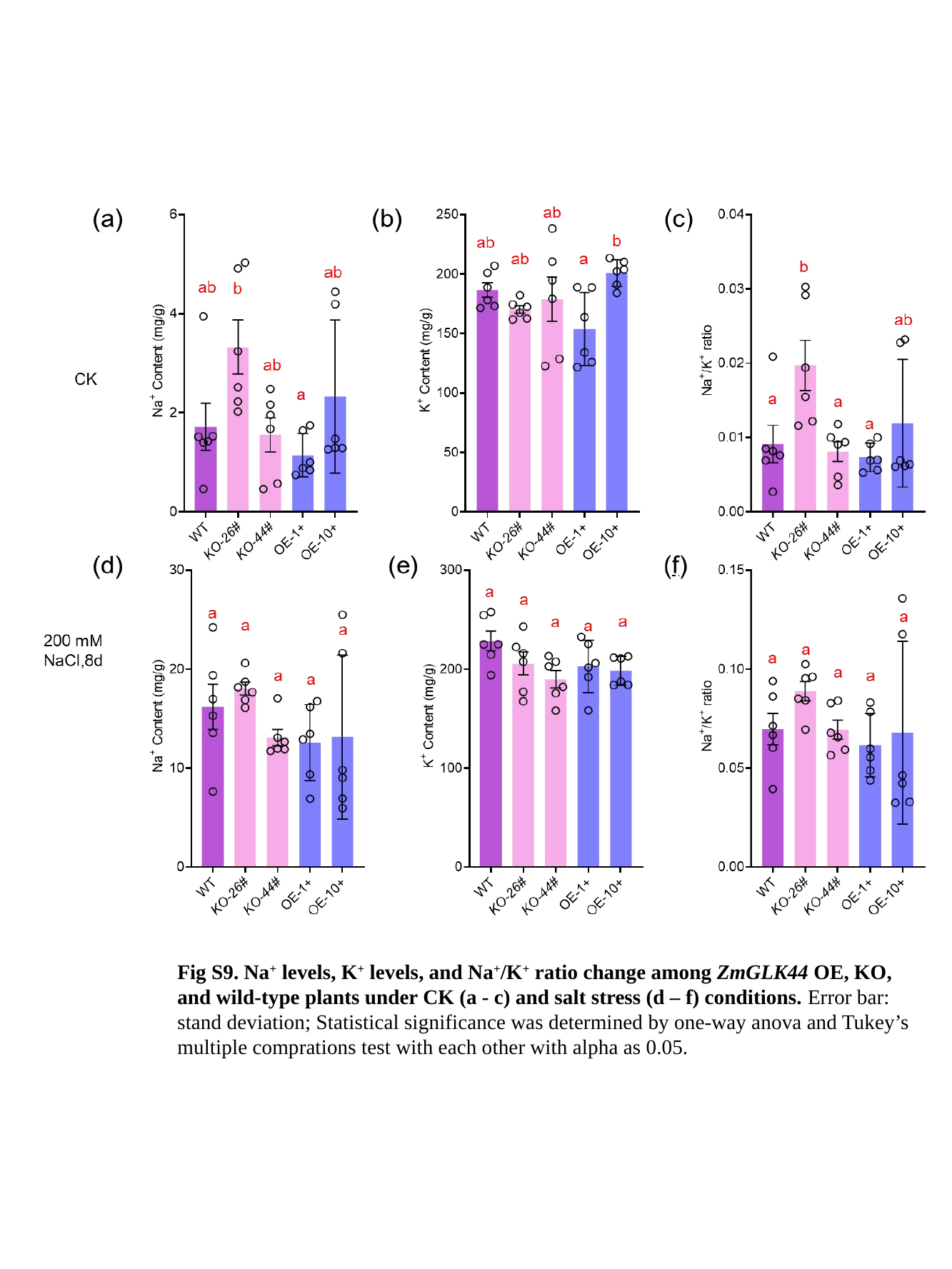

Fig S9. Na+ levels, K+ levels, and Na+/K+ ratio change among ZmGLK44 OE, KO, and wild-type plants under CK (a - c) and salt stress (d – f) conditions. Error bar: stand deviation; Statistical significance was determined by one-way anova and Tukey’s multiple comprations test with each other with alpha as 0.05.

## Slide 10
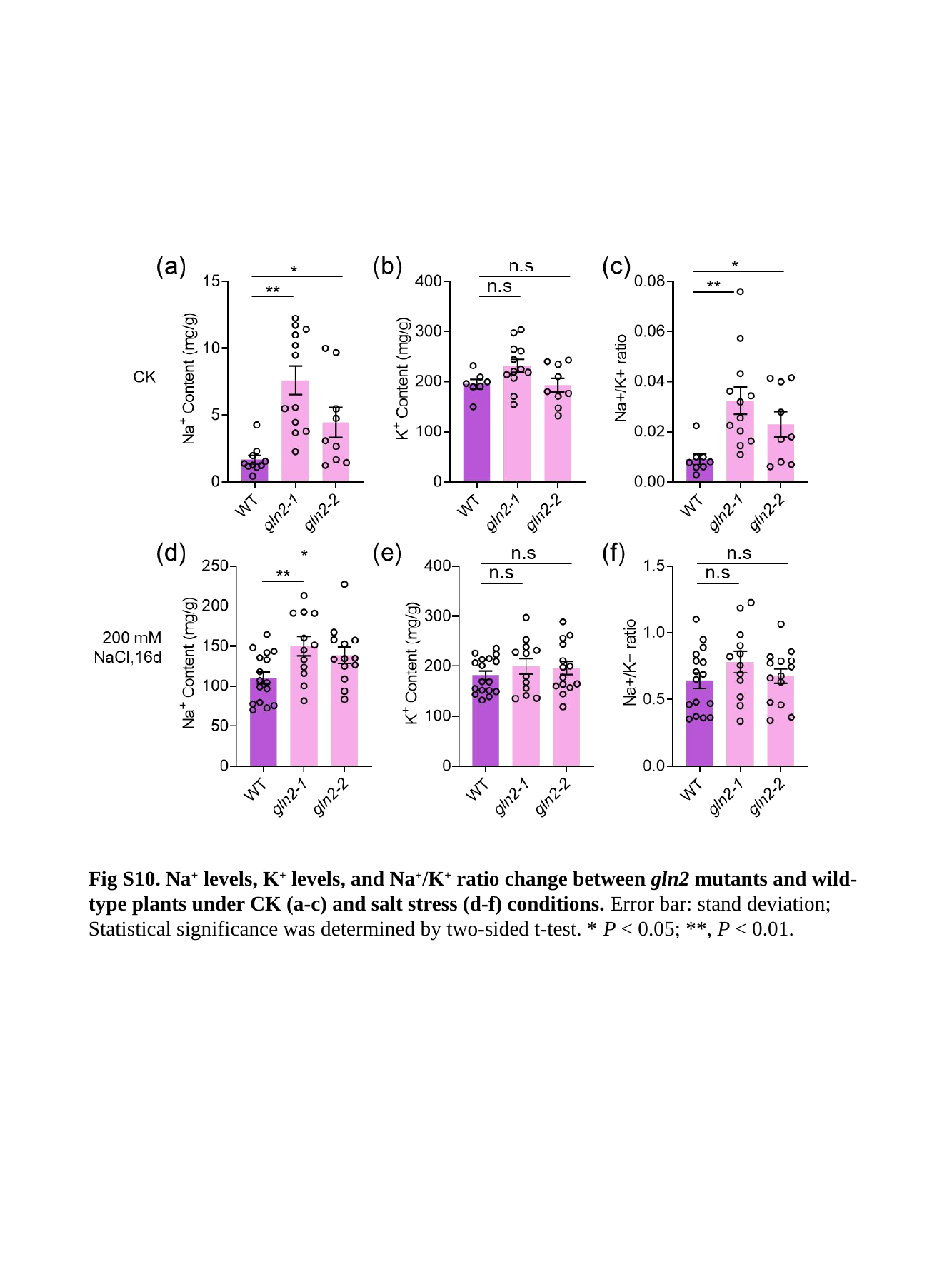

Fig S10. Na+ levels, K+ levels, and Na+/K+ ratio change between gln2 mutants and wild-type plants under CK (a-c) and salt stress (d-f) conditions. Error bar: stand deviation; Statistical significance was determined by two-sided t-test. * P < 0.05; **, P < 0.01.

## Slide 11
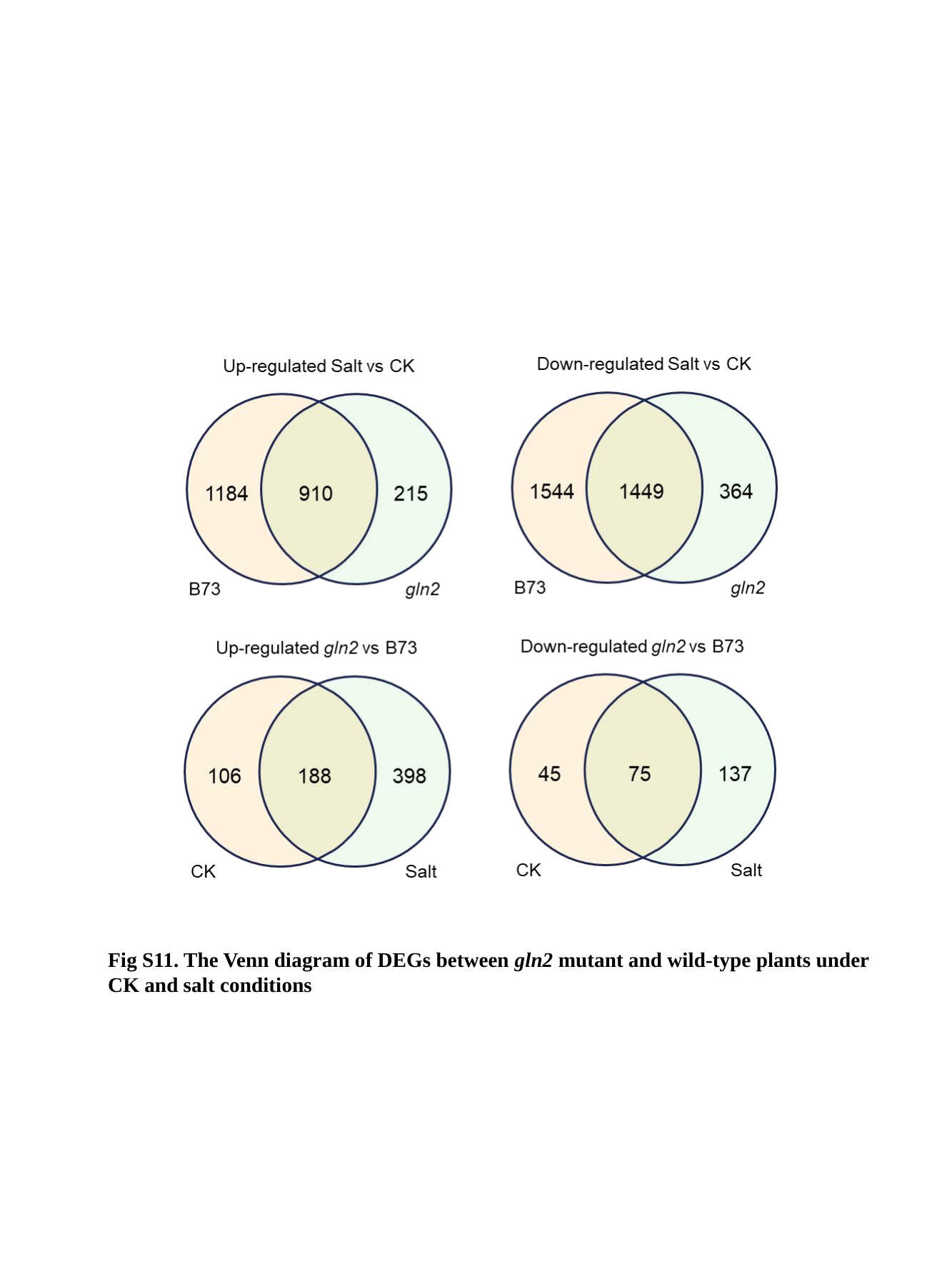

Fig S11. The Venn diagram of DEGs between gln2 mutant and wild-type plants under CK and salt conditions

## Slide 12
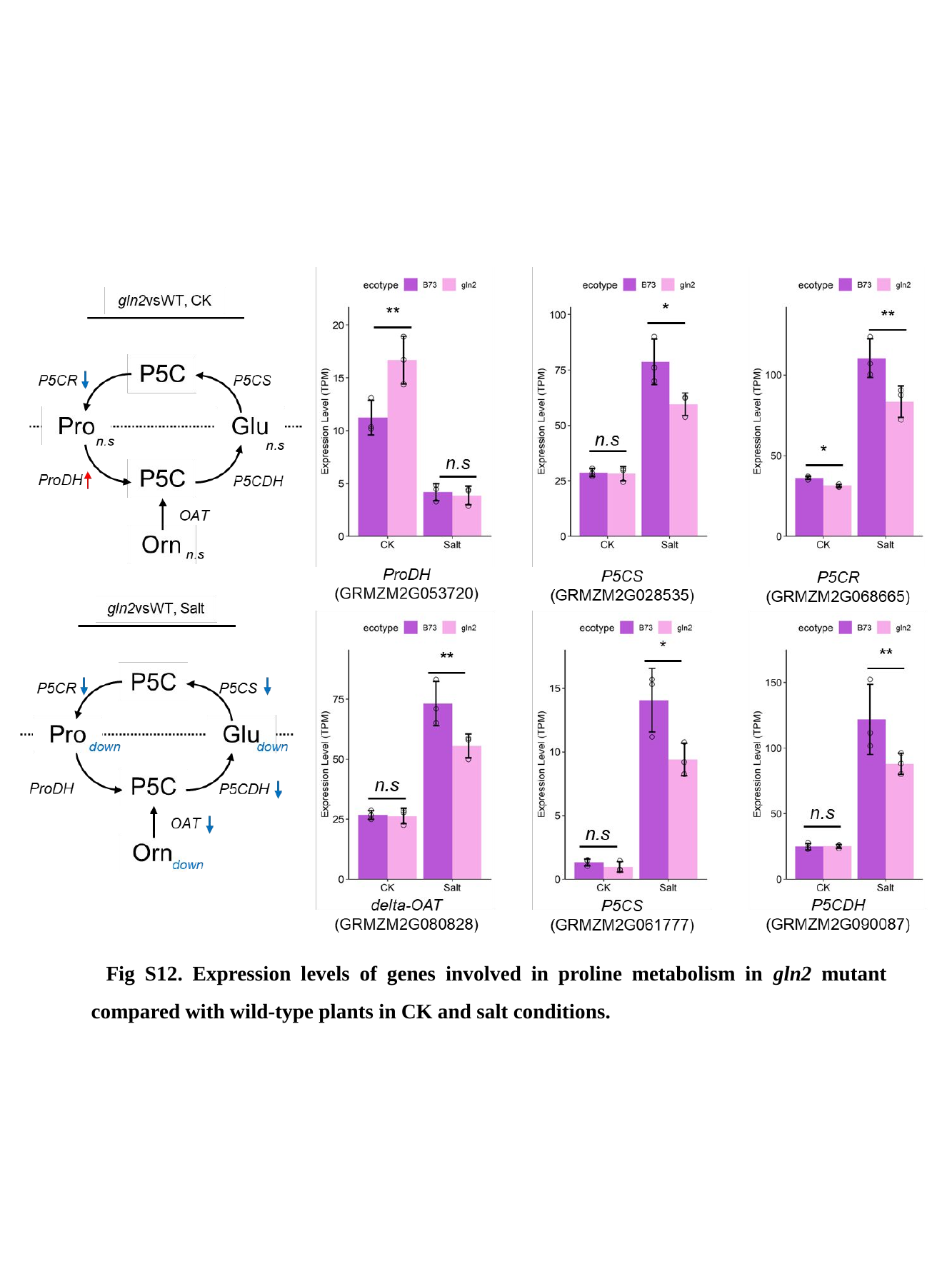

Fig S12. Expression levels of genes involved in proline metabolism in gln2 mutant compared with wild-type plants in CK and salt conditions.
